# Mechanism of Sanhua Decoction in the Treatment of Ischemic Stroke Based on Network Pharmacology Methods and Experimental Verification

**DOI:** 10.1155/2022/7759402

**Published:** 2022-01-21

**Authors:** YingHuang, Shan-shan Gao, Zi-han Gong, Wen-jie Li, Xiao-junGou, Ji-jia Sun, Ming-jie Sun

**Affiliations:** ^1^Experimental Research Center, China Academy of Chinese Medical Sciences, Beijing Key Laboratory of Research of Chinese Medicine on Prevention and Treatment for Major Diseases, Beijing 100700, China; ^2^Central Laboratory, Baoshan District Hospital of Integrated Traditional Chinese and Western Medicine of Shanghai, Shanghai University of Traditional Chinese Medicine, Shanghai 201999, China; ^3^Institute of Basic Theory for Chinese Medicine, China Academy of Chinese Medical Sciences, Beijing 100700, China; ^4^Mathematics and Physics Teaching and Research Section, School of Pharmacy, Shanghai University of Traditional Chinese Medicine, Shanghai 201203, China

## Abstract

**Objective:**

The mechanism of action of Sanhua Decoction (SHD) in the treatment of ischemic stroke (IS) was analyzed based on the network pharmacology technology, and the pharmacodynamics and key targets were verified using the rat middle cerebral artery occlusion (MCAO) model.

**Methods:**

The GEO database was used to collect IS-related gene set *S*_*D*_, and DrugBank and TTD databases were used to obtain the therapeutic drug target set *S*_*T*_. IS disease gene set *S*_*I*_ was collected from DisGeNET, GeneCards, and OMIM databases. These three different gene sets obtained from various sources were merged, duplicates were removed, and the resulting IS disease gene set *S*_IS_ was imported into the STRING database to establish the protein-protein interaction (PPI) network. Two methods were used to screen the key targets of IS disease based on the PPI network analysis. The TCMSP database and PubChem were applied to retrieve the main chemical components of SHD, and the ACD/Labs software and the SwissADME online system were utilized for ADMET screening. HitPick, SEA, and SwissTarget Prediction online systems were used to predict the set of potential targets for SHD to treat IS. The predicted set of potential targets and the IS disease gene set were intersected. Subsequently, the set of potential targets for SHD treatment of IS was identified, the target information was confirmed through the UniProt database, and finally, the component-target data set for SHD treatment of IS was obtained. clusterProfiler was used for GO function annotation and KEGG pathway enrichment analysis on the target set of SHD active ingredients. A rat MCAO model was established to evaluate the pharmacodynamics of SHD in the treatment of IS, and Western blot analysis assessed the level of proteins in the related pathways.

**Results:**

This study obtained 1,009 IS disease gene sets. PPI network analysis identified 12 key targets: AGT, SAA1, KNG1, APP, GNB3, C3, CXCR4, CXCL12, CXCL8, CXCL1, F2, and EDN1. Database analyses retrieved 40 active ingredients and 47 target genes in SHD. The network proximity algorithm was used to optimize the six key components in SHD. KEGG enrichment showed that the signaling pathways related to IS were endocrine resistance, estrogen, TNF signal pathway, and AGEs/RAGE. Compound-disease-target regulatory network analysis showed that AKT1, IL-6, TNF-*α*, TP53, VEGFA, and APP were related to the treatment of IS with SHD. Animal experiments demonstrated that SHD significantly reduces the neurological function of rat defect symptoms (*P* < 0.05), the area of cerebral avascular necrosis, and neuronal necrosis while increasing the levels of IL-6 and APP proteins (*P* < 0.05) and reducing the levels of AKT1 and VEGFA proteins (*P* < 0.05).

**Conclusion:**

The effective components of SHD may regulate multiple signaling pathways through IL-6, APP, AKT1, and VEGFA to reduce brain damage and inflammatory damage and exert a neuroprotective role in the treatment of IS diseases. Thus, this study provides a feasible method to study the pharmacological mechanism of traditional Chinese medicine compound prescriptions and a theoretical basis for the development of SHD into a new drug for IS treatment.

## 1. Introduction

Stroke is the second leading cause of death worldwide and is becoming a serious medical problem in developing countries [1, 2]. In China, approximately 1.3 million people suffer from stroke each year, and about 80% are related to ischemia [3]. Ischemic stroke (IS) is a common type of cerebrovascular disease with high morbidity and disability [4]. It is the first of the three major causes of death in China, accounting for about 50–70% of cerebrovascular accident diseases. Most surviving patients have severe disabilities, which casts a heavy economic burden on the family and society [5]. IS refers to the stenosis or occlusion of cerebral blood vessels, leading to the blockage of cerebral blood flow, which in turn causes ischemia, hypoxia, softening, and even necrosis of brain tissue, thereby resulting in cerebrovascular dysfunction and irreversible brain damage [6, 7]. Presently, the accepted theories about the pathogenesis of IS include energy exhaustion, depolarization of the penumbra around the infarct focus, excitatory amino acid (EAA) toxic effects, nitric oxide, inflammatory cytokines, free radical damage and cell damage, and apoptosis [8]. Therefore, adopting active and effective methods to start the protective program of cerebral ischemia injury in the early stage to reduce the neurological deficit and restore and reconstruct the cerebral neurological function is the current hotspot for research [9]. Clinically, patients with IS are treated with platelet aggregation inhibitors, statins, antihypertensive drugs, cerebrovascular nutrients, and other drugs [10]. Although progress has been made in treating IS in the clinic, the prevention and rehabilitation of IS are still a challenge.

Traditional Chinese medicine (TCM) has a history of thousands of years in the treatment of IS [11, 12]. SHD is a representative TCM prescription for IS [13] that comes from Liu Wansu's book “Su Tong Diseases, Qi Yi Bao Ming Ji.” It is composed of *Magnolia officinalis*, *Citrus aurantium*, *Rhubarb*, and *Notopterygium* and is used for the treatment of stroke [14] even today in the modern world [15]. Previous studies have confirmed that this prescription is an optimal treatment of IS. It reduces the blood viscosity and the risk of thrombosis recurrence and increases the cerebral infarction area in patients [16]. Although the curative effect is definite, the pharmacological mechanism underlying SHD in the treatment of IS is unclear. Network pharmacology is a novel discipline based on the theory of system biology to conduct network analysis of biological systems and select specific signal nodes for multitarget drug molecular design [17]. The research field is aimed at revealing the synergy of multimolecular drugs by analyzing their regulatory effect on the disease network [18]. Network pharmacology combines system biology, omics, and computational biology to clarify the mechanism of drug action from a holistic perspective. It has the characteristics of completeness, synergy, and dynamics [19]. These characteristics are similar to those of TCM holism [20]. This method provides a new perspective for Chinese medicine's multicomponent and multitargeted therapy [21]. This method provides a system-level understanding of the effects of drugs and the complexity of diseases through network mapping and analysis. The network pharmacology method explains the mechanism of action of Chinese medicine in various diseases [22]. Therefore, the present study used network pharmacology to explore the mechanism of SHD on the effectiveness of IS treatment. A total of 40 representative compounds of SHD's four herbal medicines were screened. Subsequently, key targets were discovered using public databases and mathematical modeling calculations, and a protein-protein interaction (PPI) network was established. GO function and KEGG pathway enrichment analyses were performed. Finally, the signal pathway was verified by animal experiments. The results showed that SHD is an effective IS treatment, significantly reducing the symptoms of neurological deficits in rats, the area of avascular necrosis of brain tissue, and the amount of neuronal necrosis. Furthermore, SHD promotes the level of IL-6 and APP proteins in rats after ischemic brain injury and reduces the level of AKT1 and VEGFA proteins. These results provide a new perspective and further research direction for SHD treatment of IS. In addition, network pharmacology strategies can help researchers simplify the complex prescription system and provide novel avenues to elucidate the mechanisms underlying TCM prescriptions.

## 2. Materials and Methods

### 2.1. IS Disease Gene Collection

#### 2.1.1. IS Differential Gene Analysis Based on Gene Expression Omnibus (GEO)

In the GEO database (https://www.ncbi.nlm.nih.gov/geo/), “Ischemic Stroke” was entered to query and collect IS-related gene expression profiling chip to obtain the IS-related expression profiling data GSE22255. The data platform was GPL570 [HG-U133_Plus_2] Affymetrix Human Genome U133 Plus 2.0 Array, consisting of 20 normal and 20 IS samples.

The GSE22255 expression profile chip data file was downloaded from GEO, and the original data were preprocessed sequentially by standardization, correction, and gene name annotation using the limma package (version 3.42.2) based on the R language (version 3.6.3). Then, differentially expressed genes (DEGs) were analyzed. The IS DEG set was also screened according to *p*.value < 0.05 and |log_2_FC| > 0.5, and ultimately, the IS gene set *S*_*D*_ was obtained based on the GEO differential gene analysis.

#### 2.1.2. Collection and Collation of Data on Known IS Therapeutic Drug Targets

“Ischemic Stroke” was entered into the DrugBank (https://www.drugbank.ca/) to search for existing small-molecule drugs for the treatment of IS and obtain target information of these drugs. Similarly, “Ischemic Stroke” was entered into the Therapeutic Target Database (TTD) (http://db.idrblab.net/ttd/) to identify IS-related targets. All targets from the two databases were merged, the points sorted, and the IS target set *S*_*T*_ was queried based on the database of the known therapeutic IS drug targets.

#### 2.1.3. Collection and Sorting of IS Disease-Related Genes

Genes related to IS diseases were collected from DisGeNET (http://disgenet.org/home/), GeneCards (https://www.genecards.org/), and OMIM (https://omim.org/) databases. The IS-related genes were entered into “Ischemic Stroke,” respectively, and searched in DisGeNET, GeneCards, and OMIM. Then, the intersection was obtained using the Venny Venn diagram tool (version 2.1.0, https://bioinfogp.cnb.csic.es/tools/venny/index.html). Subsequently, the genes overlapping in two or more databases were considered as IS disease candidate genes, and thus, the IS disease gene set *S*_*I*_ based on the query of the disease database was obtained. Finally, the three different gene sets *S*_*D*_, *S*_*T*_, and *S*_*I*_ obtained from three different sources were merged, duplicated removed, and the IS disease gene set *S*_IS_ obtained for further analysis.

### 2.2. IS Disease PPI Network Construction and Key Target Discovery

#### 2.2.1. Construction of PPI Disease Network

The IS disease gene set obtained in Section 2.1 was imported into the STRING database (version 11.0, https://string-db.org/), the parameter organization was set to *Homo sapiens*, the combined score threshold was set to 0.7, and the IS disease PPI was obtained. The Gephi (version 0.9.2) software (https://gephi.org/) was used to visualize the IS disease PPI network.

#### 2.2.2. Key Target Discovery Based on PPI Network Analysis

Typically, the critical targets in the PPI network play a key role in the disease; however, several methods are used to measure the importance of the nodes in the network. In order to elucidate and explore the key targets in the IS disease network, two methods were used to screen the key IS disease targets from different perspectives in this study.

*(1) Method 1: Comprehensive Evaluation of Node Importance Based on Information Entropy Method*. Generally, the parameter indicators in the PPI network, such as degree, closeness, betweenness, eccentricity, and page rank value, can be used to evaluate the importance of the target point from different angles in the network.

Entropy was first introduced into information theory by Shannon and since then has been widely used in engineering technology, social economy, and other fields. The evaluation method based on information entropy is that the objective weight is determined according to the size of the variability of the indicator. In the present study, an improved information entropy method (IIEM) was used to comprehensively evaluate the importance value *Z* of each target on the PPI network. The computation is as follows:

1. The network index value *x*_*ij*_ of each protein target in the PPI network is standardized and preprocessed using the following equation (1):

(1) $$\begin{array}{c} y_{ij}=\frac{x_{ij}-\min\left(x_{ij}\right)}{\max\left(x_{ij}\right)-\min\left(x_{ij}\right)}. \end{array}$$

(2) The information entropy value *H*_*j*_ of each network index is calculated using the following equation (2):

(2) $$\begin{array}{c} H_{j}=-\frac{1}{\ln m}\overset{m}{\underset{i=1}{\sum }}b_{ij}\times \ln b_{ij}. \end{array}$$

Among them, *b*_*ij*_ = (*y*_*ij*_ + *ε*)/(∑_*i*=1_^*m*^(*y*_*ij*_ + *ε*)), *ε* = 10^−4^.

(3) The weight value *ω*_j_ of each network index is calculated using the following equation (3):

(3) $$\begin{array}{c} \omega_{j}=\frac{1-H_{j}+0.1\times \sum_{j=1}^{n}\left(1-H_{j}\right)}{\sum_{j=1}^{n}\left[1-H_{j}+0.1\times \sum_{j=1}^{n}\left(1-H_{j}\right)\right]}. \end{array}$$

(4) The importance value *Z*_*i*_ of the *i* protein target in the PPI network is calculated using the following equation (4):

(4) $$\begin{array}{c} Z_{i}={\left[\overset{n}{\underset{j=1}{\sum }}w_{j}\left(y_{ij}-f_{j}\right)\right]}^{2}, \end{array}$$

where *m* is the number of protein targets in the network and *n* is the number of network indicators at *f*_*j*_ = 0. Herein, we first calculated the five indicators of each protein target in the IS disease PPI network, including degree, closeness, betweenness, eccentricity, and page rank. Then, according to the magnitude of the importance value *Z*_*i*_, the importance of the targets in the PPI network was sorted using IIEM, and the key IS target genes were screened out.

*(2) Method 2: Screening of Key Targets Based on Network Modules*. From the perspective of network modules, the critical targets in the PPI network were analyzed and screened. The CytoHubba tool in the Cytoscape (version 3.7.1) software (https://cytoscape.org/) based on the Maximal Clique Centrality (MCC) algorithm was employed, and the crucial nodes in the network were discovered from the mining perspective of the largest group of network modules.

### 2.3. Collection of the Main Ingredients and Screening of ADMET in SHD

SHD is mainly composed of four TCMs, including *Rhubarb* (DH), *Magnolia officinalis* (HP), *Citrus aurantium* (ZS), and *Notopterygium* (QH). First, we collected all the ingredients of these medicines from our TCMID database (http://www.megabionet.org/tcmid/). Then, according to the PubChem database (https://pubchem.ncbi.nlm.nih.gov), the compound name, molecular formula, PubChem CID, Canonical SMILES, and other information on each component were collected and confirmed to establish the SHD component database.

In this study, we used two ADMET prediction systems, the ACD/Labs software and SwissADME online system (http://www.swissadme.ch/), to screen the active ingredients according to the molecular weight (MW), Lipinski, solubility, bioavailability (dose = 50 mg), Ames, hERG, synthetic accessibility, and other indicators. Among these, the evaluation result of MW and Lipinski was “Good” or “Moderate,” that of solubility removal was considered as “Highly Insoluble,” the oral availability bioavailability was ≥60%, Ames and hERG were genotoxicity and cardiotoxicity indicators, respectively, the clear toxic components were removed during screening, and synthetic accessibility was ≤5.

### 2.4. Prediction and Identification of Potential Targets of Active Ingredients

Canonical SMILES corresponding to the SHD active ingredients filtered by ADMET were imported into HitPick (http://mips.helmholtz-muenchen.de/proj/hitpick), SEA (http://sea.bkslab.org/), and SwissTargetPrediction (http://www.swisstargetprediction.ch/) to predict the potential target points of all components. Among these, the screening threshold precision predicted by HitPick was 70%, the screening threshold max Tc predicted by SEA was 0.7, and the SwissTarget Prediction threshold probability was 0.6.

Next, the predicted potential target set and IS disease gene set were intersected, and the potential target set of SHD treatment of IS was identified. The target information was confirmed through the UniProt database (https://www.uniprot.org/). Finally, the component-target data of the treatment of IS by SHD was obtained. The Cytoscape software was used to draw the effective component-target network diagram of the SHD treatment of IS. This network was termed as the SHD active component direct action target network for subsequent analysis.

### 2.5. GO Function of the Target and the Enrichment Analysis of the KEGG Pathway

Cluster Profiler (version 3.14.3) was used to perform GO function annotation and KEGG pathway enrichment analysis of the target set of SHD active ingredients. The gene annotation and the signaling pathway catalogs were selected according to the *P* value < 0.05 and *q* value < 0.05 criteria. A data file was created based on the results of KEGG pathway enrichment analysis, and the target-enrichment pathway network was constructed using the Cytoscape software.

### 2.6. Regulation Analysis of Key Targets of SHD Intervention Based on the Proximity of Human PPI Networks

Next, we investigated whether the active ingredients of SHD have direct or indirect regulatory effects on the key targets in Section 2.2. Although some ingredients do not directly affect, they may influence and regulate the key targets through adjacent intervention targets. Therefore, we proposed an in-depth analysis of the effective ingredients in SHD based on the calculation method of the network proximity of the human PPI background network using equations (5) and (6):

(5) $$\begin{array}{c} S_{AB}=\langle d_{AB}\rangle -\frac{\langle d_{AA}\rangle +\langle d_{BB}\rangle }{2}, \end{array}$$

(6) $$\begin{array}{c} \langle d_{AB}\rangle =\frac{1}{\left\|A\right\|\times \left\|B\right\|}\underset{a\in A,b\in B}{\sum }d\left(a,b\right). \end{array}$$

*A* is the component action target set, ‖*A*‖ is the number of the target set, *B* is the key gene set, ‖*B*‖ is the number of the target in the key gene set, *d*(*a*, *b*) is the shortest path distance between two nodes in the human PPI background network, 〈*d*_*AA*_〉 represents the average distance between the target points of the component action, 〈*d*_*BB*_〉 represents the average distance between key genes, and  〈*d*_*AB*_〉 represents the average distance between the target set and the key target set of the component action on the background network; the network distance is calculated using the igraph package (version 1.2.5).

The human PPI background network used in the calculation integrated 15 commonly used databases and focused on high-quality PPI with five types of evidence. The PPI network consisted of 16,677 proteins and 243,603 interaction correlations. This network was used as the background network to evaluate the overall regulation of the effective components of SHD decoction on the key gene set.

Typically, when *S*_*AB*_ < 0, it means that the target set *A* and the key gene set *B* of the component's action are close in the network topology, indicating that the component can regulate the key gene set *B* by intervening in the target set *A*. When *S*_*AB*_ ≥ 0, the target set *A* and the key gene set *B* of the component action are separated in the network topology, indicating that the component has no significant regulatory intervention related to the key gene set *B*. Therefore, we can further judge the presence of certain components in SHD by calculating the value of *S*_*AB*_ that might interfere with the key target set via specific target proteins in order to improve or treat the diseases.

### 2.7. Experimental Verification

#### 2.7.1. Experimental Animals

Male Sprague–Dawley (SD) rats (8-weeks-old, bodyweight 230–250 g, clean grade) were purchased from Beijing Huafukang Biotechnology Co., Ltd., license number, SCXK (Beijing) 2019-0008. All rats were bred routinely, with free water, at a room temperature of 22–24°C.

#### 2.7.2. Establishment of Animal Model

The rat middle cerebral artery occlusion model (MCAO model) was established using the thread plug method. Rats were anesthetized by intraperitoneal injection of 1% pentobarbital (35 mg/kg body weight). The limbs of the rats were fixed on the operating table, the neck skin was prepared, the drape was routinely disinfected, and a median neck incision was made. The right common carotid artery (CCA) was exposed layer-by-layer separation, and the right CCA, internal carotid artery (ICA), and external carotid artery (ECA) were carefully separated to avoid stimulating the vagus nerve. A silk thread was placed on CCA, ICA, and ECA, respectively. At 5 mm from the bifurcation of ICA and ECA, CCA was tied, ECA was ligated at the root of CCA, and the preset silk thread was pulled to block the blood flow of ICA. Then, a small cut was made at the bifurcation of ICA and CCA, the end of the threaded bolt was inserted along the incision, and the lifting line was loosened and slowly advanced 18–20 mm; the advancing was stopped when it encountered slight resistance. The ICA was ligated, and the silk thread was left to prevent the embolus from falling off. At 90 min postoperatively, the threaded plug was withdrawn from the ICA, and reperfusion was performed. In the sham operation group, the tether was inserted only 10 mm, such that it was placed in the ICA without entering the middle cerebral artery (MCA); the rest of the steps were the same as before. After the operation, the rat MCAO model was scored according to Zea-Longa's standard scoring method [4], and the animals that met the model success criteria were included in this study.

#### 2.7.3. Grouping and Administration

After the MCAO model was successfully prepared, the rats were randomly divided into the model and SHD groups (*n* = 6 rats in each group). In addition, a blank control group and a sham operation group were set up (*n* = 6 in each group). The blank control, sham operation, and model groups were given purified water, while the SHD group was given SHD water decoction consisting of 20 g of *Rhubarb* (HD), 20 g of *Magnolia officinalis* (HP), 20 g of *Citrus aurantium* (ZS), and 20 g of *Notopterygium* (QH). It was decocted in water at a concentration of 0.864 g/mL of the crude drug. The dosage for rats was converted according to the daily clinical dosage of the patient and administered at 10 a.m. daily; each rat was administered 7.2 g/kg for 5 consecutive days.

#### 2.7.4. Neurological Deficit Score (NDS) to Detect the Recovery of the Neurological Function

Zea-Longa scoring method was used to evaluate the NDS: 0 points to without neurological deficits and normal activity, 1 point to those who could not fully extend the contralateral front paw, 2 points to those who turn to the left while crawling, 3 points to those who fall to the hemiplegic side while walking, 4 points to those who cannot walk spontaneously and have a consciousness disorder.

#### 2.7.5. 2,3,5-Triphenyltetrazolium Chloride (TTC) Staining to Detect the Cerebral Infarction Rate

At 24 h after the last administration, the rats were anesthetized with 1% pentobarbital (35 mg/kg body weight) by intraperitoneal injection to perform a craniotomy. The brain tissue was excised, and the coronal sections were cut into six slices, each about 2 mm thick. The sections were immersed in 2% TTC solution for staining. The Image-Pro Plus image analysis software was used to calculate the percentage of the white part (this is the stroke infarct part) in the cerebral hemisphere after staining. Cerebral infarction rate = (volume of the contralateral ischemic hemisphere − volume of noninfarcted ischemic side)/volume of contralateral ischemic hemisphere × 100%.

#### 2.7.6. Effect of SHD on the Pathomorphology of Cerebral Ischemia-Reperfusion Rats

The brain tissue was fixed in 4% paraformaldehyde, routinely dehydrated, paraffin-embedded, sectioned, hematoxylin-eosin (HE) stained, mounted, and placed under a Leica optical microscope to observe the morphological changes.

#### 2.7.7. Western Blot Detection

An equivalent of 100 mg of the tissue was homogenized in 1 mL of RIPA buffer using a tissue grinder (60HZ, 90S). The supernatant was collected by centrifugation of the lysate at 12000 rpm 4°C for 10 min. The total protein concentration was estimated using the BCA method. An equivalent of protein was resolved on SDS-PAGE and transferred to membrane. After blocking in TBST, the membrane was probed with AKT1, IL-6, TP53, TNF-*α*, VEGFA, and APP primary antibodies at 4°C, overnight, followed by incubation with secondary antibody at room temperature for 1 h. Subsequently, the immunoreactive bands were developed using the ECL reagent, and the protein expression level was analyzed by the Quantity One software.

#### 2.7.8. Statistical Analysis

The experimental data are expressed as mean ± standard deviation (^−^x ± sd) and analyzed using the SPSS 19 statistical software. One-way analysis of variance (ANOVA) is used to compare the mean values of samples in multiple groups. The measurement data of the two groups were evaluated by *t*-test, and *P* < 0.05 indicated statistical significance.

## 3. Results

### 3.1. Gene Collection of IS Disease

**Figure 1:**
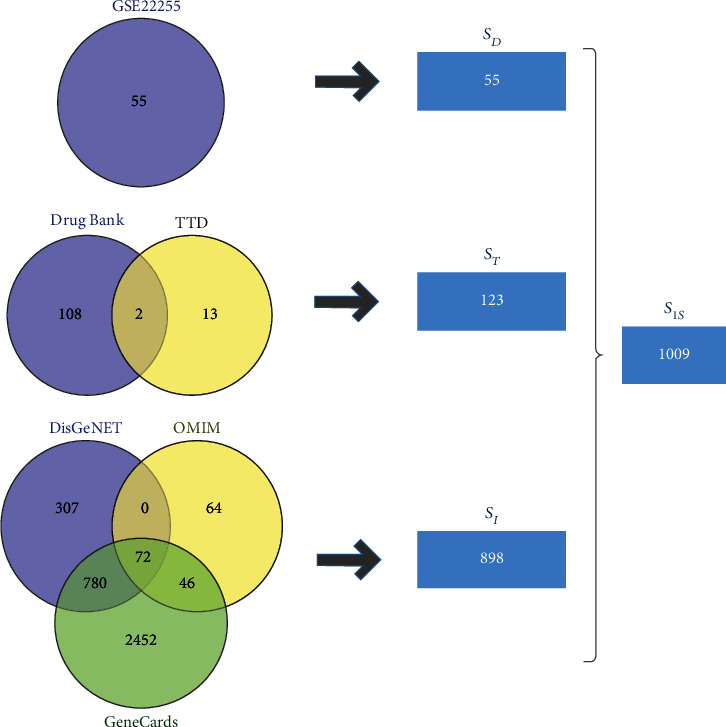
(a) 55 differentially expressed genes obtained from the GSE22255 chip GEO differential gene analysis. 123 drug treatment targets based on the query and combination of DrugBank and TTD databases. 898 genes based on the query and combination of DisGeNET, GeneCards, and OMIM databases. A total of 1009 IS disease genes were obtained by three methods.

A total of 55 DEGs related to IS disease were screened based on GEO differential gene analysis. Among these, 1 upregulated gene and 54 downregulated genes comprised the IS gene set *S*_*D*_. Subsequently, 86 small-molecule drugs for the treatment of IS and 110 targets of their effects were collected from DrugBank, and 15 related targets were obtained from TTD. Thus, a total of 123 therapeutic drug targets for IS were obtained from the combination of the two, namely, the IS target point set *S*_*T*_, based on a database of known therapeutic IS drug targets. Finally, 1159, 3350, and 182 related genes were collected from DisGeNET, GeneCards, and OMIM, respectively. All genes that overlapped in the two databases were included, and a total of 898 genes were obtained, which comprised the IS disease gene set *S*_*I*_ based on the disease data queries. Finally, the three sets of genes were merged to obtain a total of 1009 IS disease genes, i.e., the IS disease gene set *S*_IS_. The result is shown in Figure 1.

### 3.2. IS Disease PPI Network Construction and Key Target Discovery

**Figure 2:**
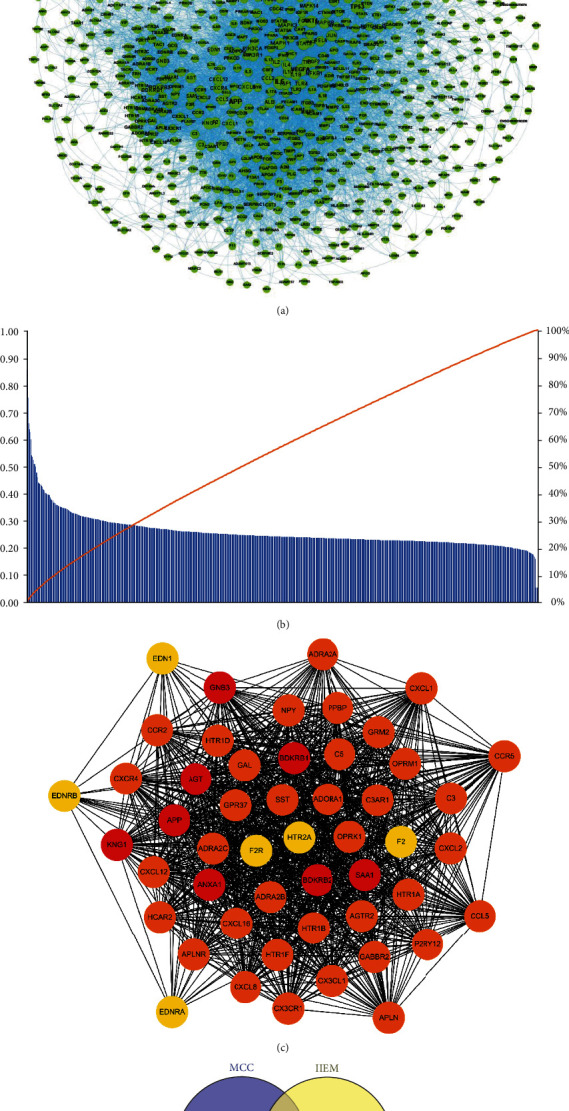
(a) PPI network of IS disease genes by STRING. (b) Pareto distribution diagram of the scores of important nodes in the PPI network based on the IIEM. (c) The results of the top 40 important targets of the PPI network based on MCC. (d) 12 key genes of IS obtained through MCC and IIEM.

All 1009 genes of the IS gene set were imported into STRING, and the PPI network of IS disease was generated using Gephi (Figure 2). Next, we calculated the importance of all nodes in the PPI network (Supplementary materials 1) based on the IIEM method. In this study, we stipulated that nodes with an evaluation score of *Z*_*i*_ ≥ 0.4 were included as the key targets, and 39 IS disease targets were obtained. We also screened and ranked the top 50 targets based on the MCC algorithm of CytoHubba. Finally, the intersection of the two methods revealed 12 key targets (AGT, SAA1, KNG1, APP, GNB3, C3, CXCR4, CXCL12, CXCL8, CXCL1, F2, and EDN1) (Table 1).

### 3.3. Collection of SHD and ADMET Screening

A total of 328 components were collected from TCMID, of which 84 were Rhubarb (DH), 20 were *Magnolia officinalis* (HP), 193 were *Notopterygium* (QH), and 31 were *Citrus aurantium* (ZS). Furthermore, the chemical information of all small molecules was collected and confirmed from the PubChem database. In addition, for the convenience of follow-up analysis, we sequentially numbered the components MOL001–MOL328. The ACD/Labs software and the SwissADME were used for ADMET screening, and 147 active ingredients meeting ADMET conditions were obtained (Supplementary materials 2).

### 3.4. Prediction and Identification of Effective Component Targets

**Figure 3:**
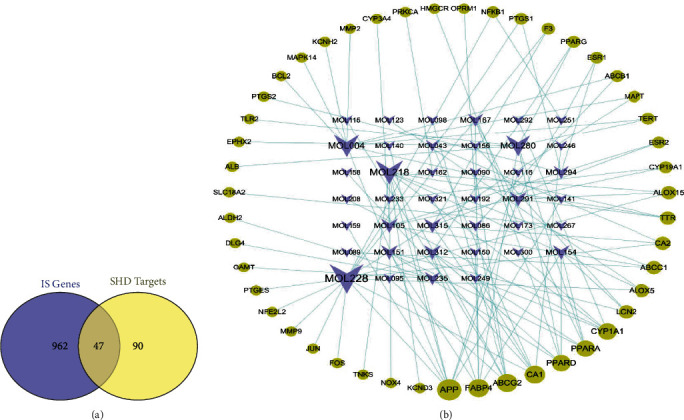
(a) Venn diagram of target prediction and recognition results of SHD. (b) SHD ingredients-direct acting target network diagram, where the yellow nodes represent the target and the V-shaped nodes represent the ingredients. The larger the node, the greater the degree value of the node in the network.

HitPick, SEA, and SwissTarget prediction databases were used to predict and filter the 147 active ingredients of SHD based on the threshold; consequently, 72 active ingredients and 137 potential targets of their effects were intersected with the IS disease gene set *S*_IS_, and it was found that the 40 active ingredients in SHD directly acted on the 47 IS targets. The information of these 47 targets is listed in Table 2. Finally, Cytoscape was used to construct the active ingredient-target direct action network of SHD (Figure 3).

### 3.5. GO Functional Annotation and KEGG Pathway Enrichment Analysis of SHD Targets

**Figure 4:**
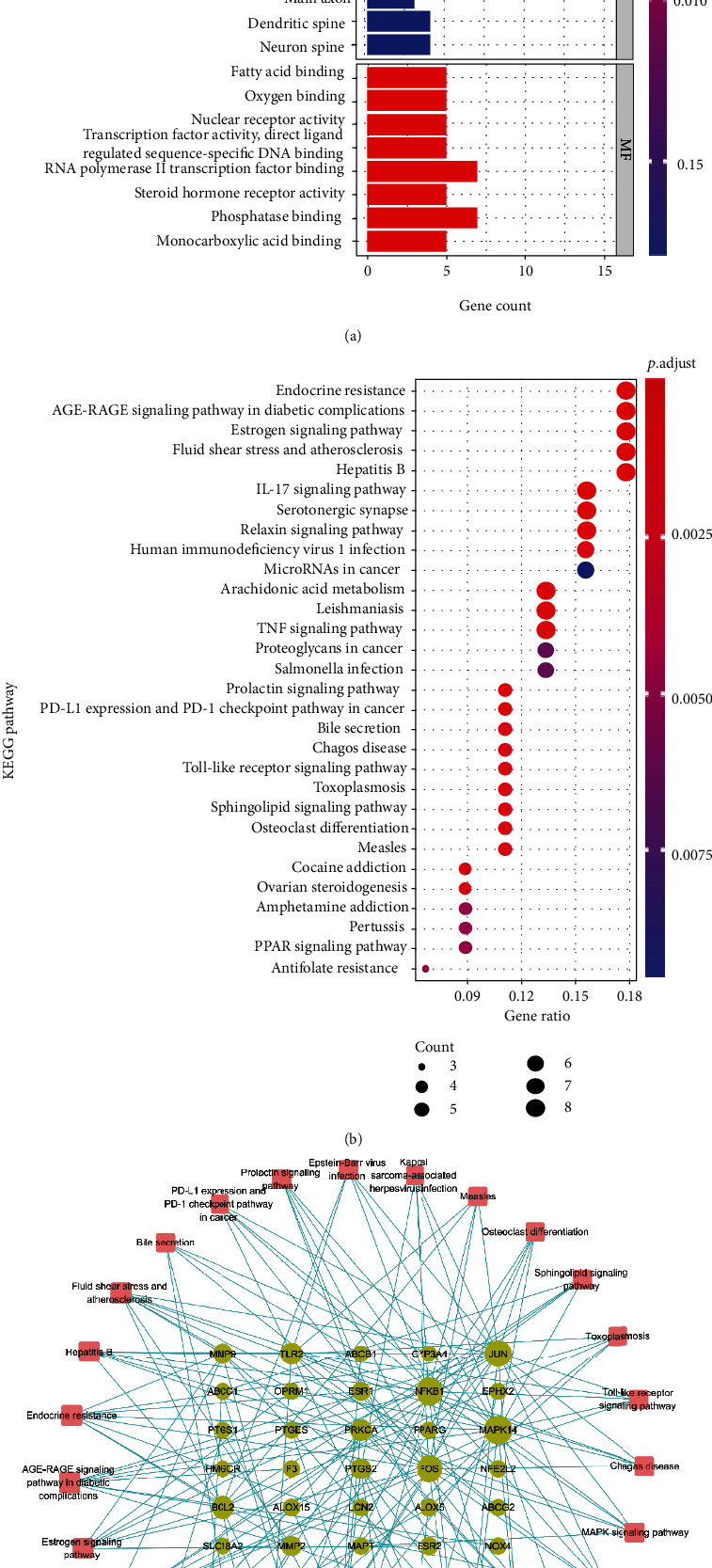
(a) GO function annotation results for the active ingredients of SHD acting on IS targets. (b) KEGG pathway enrichment analysis results for the active ingredients of SHD acting on IS targets. (c) Target-pathway enrichment network; dots represent protein targets, and squares represent pathways. The greater the degree of the node, the larger the node.

GO functional annotation and KEGG pathway enrichment analyses of 47 IS disease targets affected by SHD were performed, and the results are shown in Figures 4(a) and 4(b).

GO function annotation and KEGG pathway enrichment analyses revealed that the 47 targets are involved in 683 biological process (BP) functions, such as response to metal ion (GO: 0010038), icosanoid metabolic process (GO: 0006690), response to lipopolysaccharide (GO: 0032496), cellular response to metal ion (GO:0071248), and response to molecule of bacterial origin (GO: 0002237); 67 molecular function (MF) functions, such as fatty acid binding (GO: 0005504), oxygen binding (GO: 0019825), nuclear receptor activity (GO: 0004879), transcription factor activity, direct ligand regulated sequence-specific DNA binding (GO: 0098531), and RNA polymerase II transcription factor binding (GO: 0001085); and 21 cellular component (CC) functions, such as secretory granule lumen (GO: 0034774), cytoplasmic vesicle lumen (GO: 0060205), vesicle lumen (GO: 0031983), RNA polymerase II transcription factor complex (GO: 0090575), and nuclear transcription factor complex (GO: 0044798).

The KEGG pathway was mainly enriched in 61 signaling pathways, such as endocrine resistance (hsa01522), AGE-RAGE in diabetic complications (hsa04933), estrogen signaling pathway (hsa04915), fluid shear stress and atherosclerosis (hsa05418), Hepatitis B (hsa05161), IL-17 (hsa04657), serotonergic synapse (hsa04726), relaxin signaling pathway (hsa04926), human immunodeficiency virus 1 infection (hsa05170), and microRNAs (miRNAs) in cancer (hsa05206). The pathways with the number of enrichment targets ≥ 5 were selected, and the target-enrichment pathway network was constructed using Cytoscape (Figure 4(c)).

### 3.6. Analysis of the Regulation of Key Target Genes by Small Molecules

**Figure 5:**
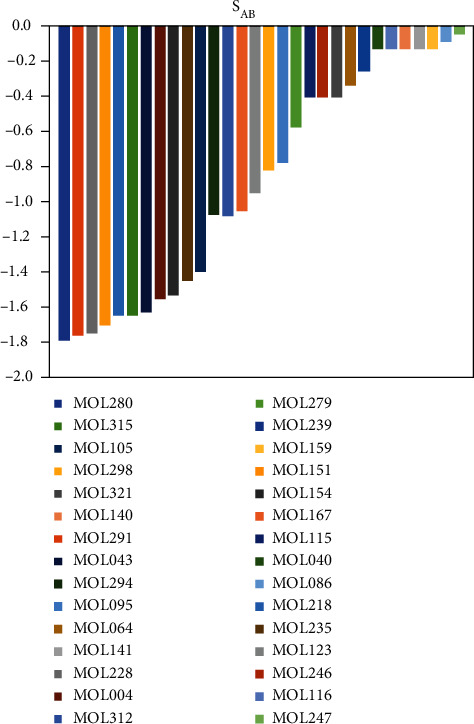
Analysis results of SHD intervention on key targets of IS based on network proximity.

According to the results in Section 2.2, we identified 12 key genes in the IS disease. Among the 47 targets directly affected by the 40 active ingredients of SHD, only APP belonged to these 12 key genes. Therefore, we further used the calculation method based on the network proximity to further explore whether these 40 active ingredients have an indirect regulatory effect on the key IS targets. The results are shown in Figure 5.

40/72 active ingredients of SHD had a network proximity of *S*_*AB*_ < 0 to the key target set and network proximity of *S*_*AB*_ < 0 to the key target set, indicating that these small molecules regulate these 12 key targets by intervening with specific targets. Interestingly, six components (MOL040, MOL064, MOL239, MOL247, MOL279, and MOL298) did not directly affect the 47 targets of IS but could directly interfere with the 12 key targets in the PPI network, which promoted the anti-IS disease.

### 3.7. Effect of SHD on Neurological Function Score and Cerebral Infarction Rate of MCAO Rats

**Figure 6:**
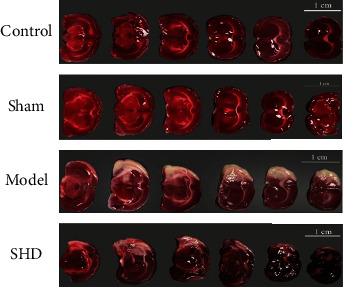
TTC brain tissue staining image.

The neurological function score results showed that the rats in the blank control and the sham-operated groups had no signs of neurological damage after the operation, and the neurological deficit score was 0. The rats in the model group showed severe symptoms of neurological impairment (*P* < 0.01). Compared to the model group, the SHD group showed significantly reduced symptoms of neurological deficit in rats (*P* < 0.05). TTC staining of nonischemic brain tissue was red, and the ischemic area was white (Figure 6). The results of TTC staining showed brain tissue infarction in the brain tissue of the model group (*P* < 0.01). Compared to the model group, the infarct area of the brain tissue in the SHD group was significantly reduced (*P* < 0.05). These results indicated that SHD reduces the neurological score of MCAO rats and the scope of cerebral infarction. The specific results are shown in Table 3.

### 3.8. Effect of IS on the Pathological Morphology of Brain Tissue in MCAO Rats

**Figure 7:**
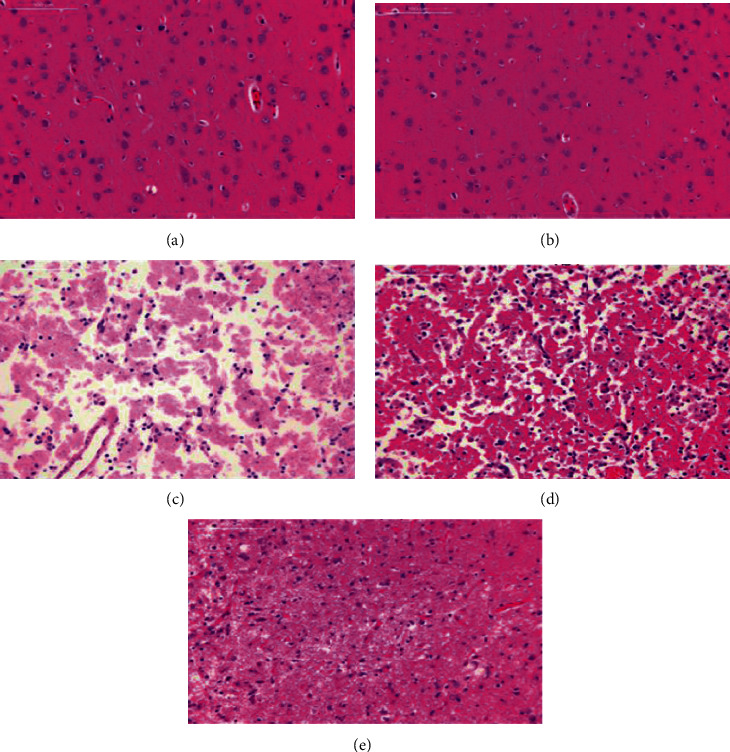
(a) Control group; (b) sham operation group; (c) model group 1; (d) model group 2; (e) SHT group.

The brain tissue of the blank and the sham operation groups was normal, the cell structure was complete, the cells were arranged neatly, the nucleus was centered, the nucleolus was clear, and the cytoplasm was not red stained. Large areas of brain tissue showed necrosis in the ischemic area of the model group, a part of the cortex showed a highly loose mesh structure, the cell structure was unclear, the number of brain tissue cells was significantly reduced, the neurons showed degeneration, necrosis, and nuclear pyknosis, nucleoli disappeared, and the cytoplasm stained red. Compared to the model group, the number of necrotic foci in the SHD group was significantly reduced, the cell arrangement was more orderly, the brain tissue structure on the ischemic side was significantly improved, and the inflammatory cell infiltration was lighter (Figure 7).

### 3.9. Western Blot Results of Brain Tissue AKT1, IL-6, TP53, TNF-*α*, VEGFA, and APP Proteins

**Figure 8:**
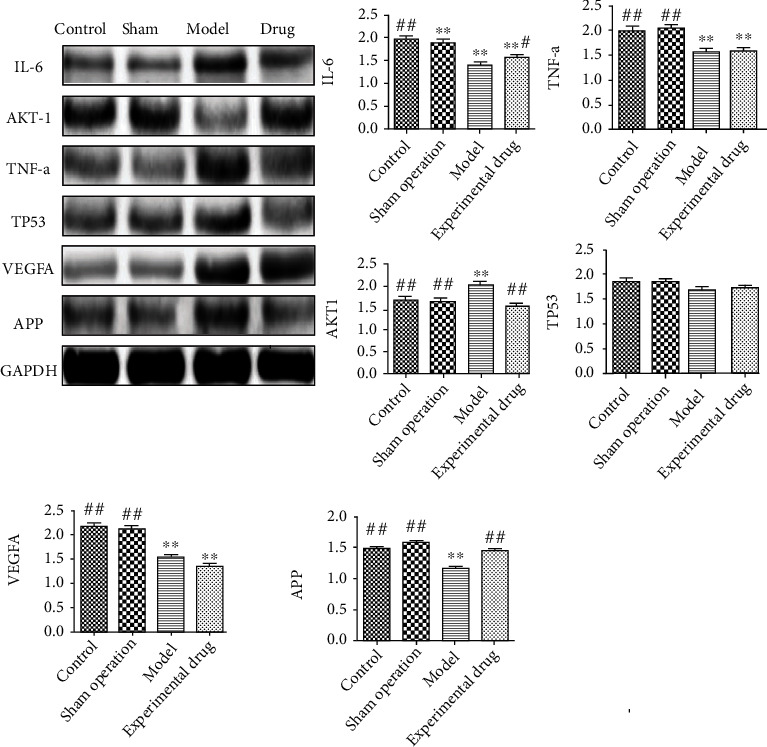
Comparison of protein expression levels in brain tissue of rats in each group.

Western blot results showed no significant difference in the brain tissue protein levels between the blank and the sham operation groups (*P* > 0.01). Compared to the sham operation group, the protein levels of the brain tissue in the model group increased except for AKT1, while the levels of other protein levels decreased (*P* < 0.01). Compared to the model group, the expression levels of TP53 and TNF-*α* proteins in the SHD group differed significantly (*P* > 0.01), the expression levels of AKT1 and VEGFA proteins decreased, and the expression levels of IL-6 and APP proteins increased (*P* < 0.05). The above results suggested that SHT drugs can promote the levels of IL-6 and APP proteins in rats after ischemic brain injury and reduce the levels of AKT1 and VEGFA proteins (Table 4 and Figure 8).

## 4. Discussion

IS is one of the leading causes of death and disability worldwide; however, currently, there is a lack of effective treatment methods [23]. The rise in alternative medicine has provided new strategies for IS treatment, especially in developing countries, where cheap and easily available Chinese herbal medicines are a major choice for patients [24]. TCM represented by compound prescriptions has accumulated a large amount of clinical practice in the treatment of IS and formed effective prescriptions [25].

SHD is a classic Chinese herbal medicine for the treatment of stroke. It is mainly composed of four TCMs, including *Rhubarb* (DH), *Magnolia officinalis* (HP), *Citrus aurantium* (ZS), and *Notopterygium* (QH), and has the functions of dispersing qi, moving blood, dredging the fu-organs, and opening up nodules, regulating qi movement and unblocking sweat pores [26]. SHD improves the NIHSS score and Glasgow score of IS patients [27] and reduces the cerebral infarction volume of focal I/R rats [28], significantly reducing and improving cerebral edema in focal cerebral I/R rats with nervous system defects [29]. However, the exact pharmacological mechanism of SHD in the treatment of IS remains unclear. The present study used network pharmacology methods to describe the correlation between active compounds, compound targets, and signaling pathways and in vivo experimental verification to reveal the mechanism underlying SHD.

In this study, 40 active compounds and 47 direct-acting target genes in SHD were identified, indicating that SHD plays a pharmacological role in treating IS through multiple targets. Emodin anthrone, Isoimperatorin, and Scopoletin have been identified as critical active compounds in SHD. Dyslipidemia plays a critical role in the pathogenesis of IS. The elevated cholesterol and reduced HDL levels are related to the increased risk of IS [30]. The anthraquinone derivative Emodin has a significant cholesterol-lowering effect. When Emodin enters the animal's body, it reduces the body's absorption of exogenous cholesterol, and on the other hand, it inhibits the synthesis of endogenous cholesterol in the body, thereby reducing total cholesterol and triglycerides [31]. Therefore, Emodin anthrone could be utilized to treat IS by regulating blood lipids. Emodin inhibits the activation of the MAPK-ERK pathway by downregulating ROS expression and reducing the expression of the nuclear transcription factor c-Myc and the proliferation protein Ki67. These effects inhibit the excessive proliferation of vascular smooth muscle cells (VSMCs) in the neointima and reduce the neointima membrane formation after carotid artery injury in rats, ultimately improving carotid artery stenosis, preventing and treating atherosclerosis and cardio-cerebrovascular diseases caused by carotid artery stenosis [32]. Isoperatorin is the active ingredient of notopterygium in SHD [33]. The protective effect of notopterygium extract (water extract and alcohol extract) on the brain has been verified in various cerebral ischemia and hypoxia animal models. In the hypoxia tolerance test, both extracts can improve tissue damage, reduce oxygen consumption, and prolong the survival time of mice [34]. The alcohol extract of notopterygium inhibits platelet aggregation, antithrombosis, and cerebral blood flow, which exerts a specific influence on hemorheology indexes [35]. Scopoletin has a significant protective effect on glutamate-induced neurotoxicity in HT22 cells [36]. In addition, the drug has neuron protection, reduces neuronal apoptosis, and improves neuronal autophagy, which could be attributed to the trigger of the AMPK/mTOR signaling pathway by stimulating the autophagy of the rat model induced by spinal cord injury (SCI) and improving the functional recovery of rats induced by SCI [37]. Thus, it is speculated that SHD is a multicomponent formula with multitarget therapeutic effects, and the correlation between these active compounds and IS should be studied in depth.

In this study, AKT1, IL-6, TNF-*α*, TP53, VEGFA, and APP were identified as six key protein targets related to IS. Isoimperatorin has pharmacological effects, such as analgesia, anti-inflammatory, and vasodilation [38]. Isoimperatorin inhibits the TNF-*α*-induced ROS/PI3K/Akt/NF-*κ*B signaling pathway and exerts an anti-inflammatory effect [39]. Scopoletin is a natural coumarin [40] that prevents the phosphorylation of PI3K and AKT proteins [41]. Tabana et al. [42] found that Scopoletin exerts an antiangiogenic effect by regulating VEGFA signaling. IL-6 is a proinflammatory cytokine with a low level in the central nervous system under normal conditions [43]. When brain tissue is damaged, the level of some proinflammatory cytokine TNF-*α* increases, which promotes the production of IL-6 by other inflammatory cytokines [44]. IL-6 has various proinflammatory effects that might increase the stimulation and development of early inflammatory damage in the brain and its vasculature [45]. The results of the Akhter et al. [46] study showed that IL-6 levels increased after IS, which in turn elevated the rate of cerebral infarction and worsened the clinical results. Accumulating evidence showed that the membrane protein APP has a neuroprotective effect under metabolic stress. When acute (stroke and cardiac arrest) or chronic (cerebrovascular disease) hypoxic-ischemic disease occurs, APP will be upregulated [47]. Blumenau et al. [48] showed that *APP-Aß* catabolism genes are significantly upregulated in the event of insufficient cerebral blood supply. Previous studies on network pharmacology predictions and animal model verification confirmed that SHD could treat IS diseases by promoting the expression of IL-6 and APP proteins. Animal experiments have shown that SHD significantly increases the levels of IL-6 and APP proteins and reduces the levels of AKT1 and VEGFA proteins. Therefore, we can infer that SHD achieves anti-inflammatory effects by inhibiting the expression of TNF-*α* and then inhibits the expression of AKT1 and VEGFA and effectuates IS treatment.

The results of GO enrichment analysis showed that SHD is related to major biological processes (for example, the reaction of metal ions, the metabolic process of eicosanic acid, the reaction of lipopolysaccharide, and the reaction of bacteria-derived molecules). KEGG pathway enrichment analysis showed that SHD has a therapeutic effect on IS through regulatory pathways (such as endocrine resistance signaling pathway, estrogen signaling pathway, TNF signaling pathway, AGEs/RAGE signaling pathway, and miRNAs in cancer). Metal homeostasis disorder (BMD) in the brain is considered a plausible cause of various neurodegenerative diseases [49]. The excessive concentration of divalent metal ions is a known mediator of acute IS injury [50]. Decanoic acid is a saturated fatty acid. Usually, the saturated fatty acid is a “bad” fatty acid. Saturated fatty acids increase the level of serum LDL-C, leading to cholesterol deposition in the inner arterial walls, which makes the human body susceptible to various cardiovascular diseases [51]. Lipopolysaccharide (LPS) is the main component of the cell wall of Gram-negative bacteria that mediates severe inflammation [52]. Several studies have identified four bacterial metabolic pathways, and LPS synthesis is significantly enriched in patients with IS [53]. After a stroke, the intestinal flora is disordered, and the LPS metabolite of the flora increases [54]. The LPS-mediated inflammatory response damages the intestinal barrier, and the leakage of the intestinal wall causes excessive pathogenic bacteria and LPS circulation into the blood. Subsequently, pathogenic bacteria and LPS enter the brain tissue through the damaged blood-brain barrier, aggravating brain tissue damage [55, 56]. Estrogen has neuroprotective effects in the central nervous system injuries, such as spinal cord injury, traumatic brain injury, and ischemic brain injury. Animal experiments have also shown the potential neuroprotective effects of estrogen, inhibiting the secretion of proinflammatory cytokines by microglia and astrocytes, reducing the neuroinflammatory response after cerebral ischemia through the estrogen receptor signaling pathway, and reducing neuronal death after cerebral ischemia through PI3K-Akt-GSK3 and MAPK/ERK signaling pathways [57].TNF-*α* activates microglia, promotes the adhesion and chemokine expression, and improves the migration ability of inflammatory-related cells, which is one of the key reasons for neuronal damage after IS [58]. In addition, animal studies demonstrated that the cerebral infarction volume and the degree of brain damage after cerebral ischemia in mice lacking the TNF receptor gene are significantly higher than those in wild-type mice, suggesting the neuroprotective role of TNF [59]. RAGE is a member of the immunoglobulin superfamily of cell surface molecules [60] and acts as a proinflammatory mediator in the inflammatory response [61]. AGEs are critical ligands of RAGE. They activate the microglia by acting on the receptor RAGE, induce the release of IL-1*β* and TNF-*α*, and mediate immunoinflammatory response [62]. These studies have shown that IL-1*β* and TNF-*α* play a critical role in nerve injury [63]. IS involves various BPs, including hypoxia, neuronal necrosis, and a strong inflammatory response [64, 65]. miRNAs, long noncoding RNAs (lncRNAs), and circular RNAs (circRNAs) participate in RNA-mediated networks through complex mechanisms, and these networks are related to IS [66].

The current results confirmed the neuroprotective effect of SHD in MCAO rats. Specifically, SHD reduces the neurological function score of MCAO rats, the scope of cerebral infarction, and brain tissue necrosis, followed by orderly arrangement of brain cells, improvement in the brain tissue structure of the ischemic side, and decreased infiltration of the inflammatory cells. Aquaporin-4 (AQP4) is an abundant aquaporin in the brain that regulates water transport to maintain homeostasis. Cerebral edema caused by AQP4 overexpression is the main determinant of progressive neuronal damage during cerebral ischemia [67]. SHD alleviates the neurological deficit of rats with I/R injury, reduces brain water content, and downregulates the expression of AQP4. It also has a neuroprotective effect on focal brain I/R injury in rats by targeting AQP4 [29], promoting IL-6 and APP protein expression level in rats after ischemic brain injury, and reducing the level of AKT1 and VEGFA proteins. Emodin inhibits the synthesis of inflammatory factors downstream of the NF-*κ*B pathway (TNF-*α*, IL-1, and IL-6), mediated by TLR-2 and PPAR*γ*, thereby reducing the infiltration of inflammatory cells and alleviating the inflammatory response [68]. Jiang et al. [69] found that notopterygium extract reduces the secretion of amyloid*β*-protein-40 (A*β*-40) and A*β*-42 in APPswe293T cells and inhibits the phosphorylation of GSK3*β*/tau in AKT/PKC N2a cells. In addition, chronic oral administration of notopterygium extract improves the cognitive ability of APP/PS1 mice. Many coumarin compounds in notopterygium have specific effects on the central nervous system [70]. These findings indicated that the mechanism of SHD on IS is related to the pivotal targets of IL-6, APP, AKT1, and VEGFA. Therefore, we can speculate that SHD exerts a therapeutic effect on IS through these active compounds, target genes, and signaling pathways.

## 5. Conclusion

In this study, Emodin anthrone, Isoimperatorin, and Scopoletin were identified as critical active compounds, and IL-6, APP, AKT1, and VEGFA were considered as the main targets. SHD may treat IS through signaling pathways, including endocrine resistance, estrogen, TNF, and AGEs/RAGE and microRNAs in cancer. Some studies have shown that SHD reduces the symptoms of neurological deficits in rats, the area of cerebral avascular necrosis, and the number of neuronal necrosis and has a therapeutic effect on IS, and the mechanism lies in the regulation of related target proteins. This study proves the potential pharmacological mechanism of SHD on IS and provides a reference for the clinical application of SHD.

## Figures and Tables

**Table 1:** Information table of 12 key genes.

Genes	Protein names	UniProt ID
*AGT*	Angiotensinogen	P01019
*APP*	Amyloid-beta precursor protein	P05067
*C3*	Complement C3	P01024
*CXCL1*	Growth-regulated alpha protein	P09341
*CXCL12*	Stromal cell-derived factor 1	P48061
*CXCL8*	Interleukin-8	P10145
*CXCR4*	C-X-C chemokine receptor type 4	P61073
*EDN1*	Endothelin-1	P05305
*F2*	Prothrombin	P00734
*GNB3*	Guanine nucleotide-binding protein G(I)/G(S)/G(T) subunit beta-3	P16520
*KNG1*	Kininogen-1	P01042
*SAA1*	Serum amyloid A-1 protein	P0DJI8

**Table 2:** Information table of SHD targets.

Targets	Protein names	UniProt ID
ABCB1	ATP-dependent translocase ABCB1	P08183
ABCC1	Multidrug resistance-associated protein 1	P33527
ABCG2	Broad substrate specificity ATP-binding cassette transporter ABCG2	Q9UNQ0
ALB	Albumin	P02768
ALDH2	Aldehyde dehydrogenase	P05091
ALOX15	Polyunsaturated fatty acid lipoxygenase ALOX15	P16050
ALOX5	Polyunsaturated fatty acid 5-lipoxygenase	P09917
APP	Amyloid-beta precursor protein	P05067
BCL2	Apoptosis regulator Bcl-2	P10415
CA1	Carbonic anhydrase 1	P00915
CA2	Carbonic anhydrase 2	P00918
CYP19A1	Aromatase	P11511
CYP1A1	Cytochrome P450 1A1	P04798
CYP3A4	Cytochrome P450 3A4	P08684
DLG4	Disks large homolog 4	P78352
EPHX2	Bifunctional epoxide hydrolase 2	P34913
ESR1	Estrogen receptor	P03372
ESR2	Estrogen receptor beta	Q92731
F3	Tissue factor	P13726
FABP4	Fatty acid-binding protein	P15090
FOS	Proto-oncogene c-Fos	P01100
GAMT	Guanidinoacetate N-methyltransferase	Q14353
HMGCR	3-Hydroxy-3-methylglutaryl-coenzyme A reductase	P04035
JUN	Transcription factor AP-1	P05412
KCND3	Potassium voltage-gated channel subfamily D member 3	Q9UK17
KCNH2	Potassium voltage-gated channel subfamily H member 2	Q12809
LCN2	Neutrophil gelatinase-associated lipocalin	P80188
MAPK14	Mitogen-activated protein kinase 14	Q16539
MAPT	Microtubule-associated protein tau	P10636
MMP2	Matrix metalloproteinase-2	P08253
MMP9	Matrix metalloproteinase-9	P14780
NFE2L2	Nuclear factor erythroid 2-related factor 2	Q16236
NFKB1	Nuclear factor NF-kappa-B p105 subunit	P19838
NOX4	NADPH oxidase 4	Q9NPH5
OPRM1	Mu-type opioid receptor	P35372
PPARA	Peroxisome proliferator-activated receptor alpha	Q07869
PPARD	Peroxisome proliferator-activated receptor delta	Q03181
PPARG	Peroxisome proliferator-activated receptor gamma	P37231
PRKCA	Protein kinase C alpha type	P17252
PTGES	Prostaglandin E synthase	O14684
PTGS1	Prostaglandin G/H synthase 1	P23219
PTGS2	Prostaglandin G/H synthase 2	P35354
SLC18A2	Synaptic vesicular amine transporter	Q05940
TERT	Telomerase reverse transcriptase	O14746
TLR2	Toll-like receptor 2	O60603
TNKS	Poly [ADP-ribose] polymerase tankyrase-1	O95271
TTR	Transthyretin	P02766

**Table 3:** Effect of SHD on the neurological function score and cerebral infarction rate in MCAO rats (^−^*x* ± sd, *n* = 6).

Group	Group neurological score	Cerebral infarction rate (%)
Control	0	0
Sham	0	0
Model	1.75 ± 0.42^##^	53.67 ± 0.19^##^
SHD	1.25 ± 0.42^∗∗^	34.26 ± 0.18^∗∗^

^##^Compared to the sham operation group, *P* < 0.01. ^∗∗^Compared to the model group, *P* < 0.01.

**Table 4:** Protein levels in the brain tissue samples of rats in each group (^−^*x* ± sd, *n* = 6).

Group	AKT1	IL-6	TP53	TNF-*α*	VEGFA	APP
Control	1.68 ± 0.22^##^	1.96 ± 0.19^##^	1.85 ± 0.18	2.00 ± 0.19^##^	2.16 ± 0.21^##^	1.48 ± 0.12^##^
Sham	1.63 ± 0.16^##^	1.90 ± 0.18^∗∗^	1.83 ± 0.18	2.04 ± 0.19^##^	2.10 ± 0.20^##^	1.57 ± 0.13^##^
Model	2.01 ± 0.19^∗∗^	1.41 ± 0.12^∗∗^	1.68 ± 0.16	1.57 ± 0.15^∗∗^	1.54 ± 0.16^∗∗^	1.16 ± 0.10^∗∗^
SHD	1.53 ± 0.13^##^	1.58 ± 0.12^∗∗^^,#^	1.72 ± 0.14	1.59 ± 0.13^∗∗^	1.35 ± 0.13^∗∗^	1.45 ± 0.10^##^

^∗∗^Compared to the blank, *P* < 0.01. ^##^Compared to the model group, *P* < 0.01. ^#^Compared to the model group, *P* < 0.05.

## Data Availability

The data used to support the findings of this study are available from the corresponding author upon request.

## Supplementary Materials

Supplementary Materials Supplementary materials 1. According to the steps described in Method 1 in Section 1.2, the importance values of all nodes in the PPI network of IS disease are calculated. Supplementary materials 2 Supporting material 2 is the data of 147 active ingredients in the TCM compound obtained after screening by ADMET.
