# Supplementary material for: Mechanism of Sanhua Decoction in the Treatment of Ischemic Stroke Based on Network Pharmacology Methods and Experimental Verification
Source: Biomed Res Int. 2022 Jan 21;2022:7759402. doi: 10.1155/2022/7759402 (PMC8799339; doi:10.1155/2022/7759402)
Supplement: Supplementary Materials — 1. According to the steps described in Method 1 in Section 1.2, the importance values of all nodes in the PPI network of IS disease are calculated. Supplementary materials 2 Supporting material 2 is the data of 147 active ingredients in the TCM compound obtained after screening by ADMET. [file 7759402.f1.docx]

Supplementary materials 1

According to the steps described in Method 1 in Section 1.2, the importance values of all nodes in the PPI network of IS disease are calculated, and supporting material 1 is the scoring value of all nodes in the PPI network.

| Targets | Z |
| --- | --- |
| TP53 | 0.95 |
| IL6 | 0.86 |
| APP | 0.81 |
| AKT1 | 0.75 |
| VEGFA | 0.66 |
| TNF | 0.64 |
| MAPK1 | 0.63 |
| STAT3 | 0.60 |
| PIK3CA | 0.54 |
| KNG1 | 0.54 |
| MAPK3 | 0.54 |
| PIK3R1 | 0.53 |
| CXCL8 | 0.51 |
| MAPK8 | 0.51 |
| JUN | 0.51 |
| ALB | 0.50 |
| IGF1 | 0.48 |
| GNB3 | 0.48 |
| F2 | 0.46 |
| PTGS2 | 0.44 |
| MMP9 | 0.44 |
| IL1B | 0.44 |
| NFKB1 | 0.44 |
| AGT | 0.44 |
| IL10 | 0.43 |
| RELA | 0.43 |
| MAPK14 | 0.43 |
| EDN1 | 0.42 |
| BDNF | 0.42 |
| CXCL12 | 0.41 |
| SAA1 | 0.41 |
| IL4 | 0.40 |
| CTNNB1 | 0.40 |
| C3 | 0.40 |
| FOS | 0.40 |
| CASP3 | 0.40 |
| CXCL1 | 0.40 |
| TLR4 | 0.40 |
| CXCR4 | 0.40 |
| PPBP | 0.39 |
| JAK2 | 0.38 |
| NOS3 | 0.38 |
| CCL2 | 0.38 |
| FGF2 | 0.38 |
| RAC1 | 0.37 |
| ANXA1 | 0.37 |
| IL2 | 0.37 |
| CCL5 | 0.37 |
| SERPINE1 | 0.36 |
| VWF | 0.36 |
| APOB | 0.36 |
| TGFB1 | 0.36 |
| FGA | 0.36 |
| BDKRB2 | 0.36 |
| FGG | 0.35 |
| ICAM1 | 0.35 |
| SIRT1 | 0.35 |
| PLG | 0.35 |
| PPARG | 0.35 |
| APOE | 0.35 |
| CCR5 | 0.35 |
| NGF | 0.35 |
| CRP | 0.35 |
| SHC1 | 0.35 |
| BDKRB1 | 0.35 |
| APOA1 | 0.35 |
| HGF | 0.35 |
| TIMP1 | 0.34 |
| CDC42 | 0.34 |
| LEP | 0.34 |
| GCG | 0.34 |
| AGTR1 | 0.34 |
| ESR1 | 0.34 |
| TRAF6 | 0.33 |
| CSF2 | 0.33 |
| AGTR2 | 0.33 |
| VCAM1 | 0.33 |
| IFNG | 0.33 |
| ITGB3 | 0.33 |
| AVP | 0.33 |
| PTEN | 0.33 |
| CAT | 0.33 |
| NPY | 0.33 |
| CAV1 | 0.33 |
| IL17A | 0.33 |
| PRKCA | 0.32 |
| C3AR1 | 0.32 |
| MTOR | 0.32 |
| SERPINC1 | 0.32 |
| CXCL2 | 0.32 |
| KDR | 0.32 |
| IL13 | 0.32 |
| B2M | 0.32 |
| SMAD3 | 0.32 |
| ITGB2 | 0.32 |
| MMP2 | 0.32 |
| STAT5A | 0.32 |
| F5 | 0.32 |
| MTHFR | 0.32 |
| CCR2 | 0.32 |
| AHSG | 0.32 |
| PRKCD | 0.32 |
| DECR1 | 0.31 |
| THBS1 | 0.31 |
| CD28 | 0.31 |
| HP | 0.31 |
| TLR2 | 0.31 |
| NR3C1 | 0.31 |
| FGB | 0.31 |
| FOXO3 | 0.31 |
| PROC | 0.31 |
| SPP1 | 0.31 |
| CD40LG | 0.31 |
| IL18 | 0.31 |
| F2R | 0.31 |
| CASP8 | 0.31 |
| SST | 0.31 |
| NOTCH1 | 0.31 |
| HMOX1 | 0.31 |
| ADIPOQ | 0.31 |
| CALM1 | 0.31 |
| RETN | 0.30 |
| TAC1 | 0.30 |
| OPRM1 | 0.30 |
| STAT5B | 0.30 |
| HTR1A | 0.30 |
| A2M | 0.30 |
| DLG4 | 0.30 |
| HDAC1 | 0.30 |
| PIK3CB | 0.30 |
| C5 | 0.30 |
| CX3CL1 | 0.30 |
| P2RY12 | 0.30 |
| SYK | 0.30 |
| GRM1 | 0.30 |
| CD40 | 0.30 |
| ADRA2C | 0.30 |
| ADRA2B | 0.30 |
| PDGFRB | 0.30 |
| IRS1 | 0.30 |
| CST3 | 0.30 |
| AR | 0.30 |
| TGFB3 | 0.30 |
| HTR2C | 0.30 |
| GAL | 0.29 |
| EDNRA | 0.29 |
| NFKBIA | 0.29 |
| CX3CR1 | 0.29 |
| HTR2A | 0.29 |
| ADRA2A | 0.29 |
| NGFR | 0.29 |
| APLN | 0.29 |
| SOD2 | 0.29 |
| PPARA | 0.29 |
| TNFSF11 | 0.29 |
| TBXA2R | 0.29 |
| GRM2 | 0.29 |
| CACNA1C | 0.29 |
| APLNR | 0.29 |
| OPRK1 | 0.29 |
| IGF1R | 0.29 |
| HSPA4 | 0.29 |
| GABBR2 | 0.29 |
| ADORA1 | 0.29 |
| HTR1B | 0.29 |
| HTR1D | 0.29 |
| F8 | 0.29 |
| FOXP3 | 0.29 |
| CDKN2A | 0.29 |
| HCAR2 | 0.29 |
| HIF1A | 0.29 |
| MAPK10 | 0.29 |
| IGFBP3 | 0.29 |
| FIGF | 0.29 |
| ADRA1B | 0.29 |
| CXCL16 | 0.29 |
| MMP1 | 0.29 |
| ACTB | 0.29 |
| GPR37 | 0.29 |
| IL5 | 0.29 |
| NOS1 | 0.28 |
| HTR1F | 0.28 |
| ARG1 | 0.28 |
| EDNRB | 0.28 |
| F13A1 | 0.28 |
| P2RY1 | 0.28 |
| HCRT | 0.28 |
| APOA5 | 0.28 |
| MMP3 | 0.28 |
| FBN1 | 0.28 |
| CCL11 | 0.28 |
| ITGA2B | 0.28 |
| GSK3B | 0.28 |
| TRH | 0.28 |
| TNFRSF1A | 0.28 |
| CSF3 | 0.28 |
| IL15 | 0.28 |
| EPO | 0.28 |
| CASP1 | 0.28 |
| HSPG2 | 0.28 |
| GNAS | 0.28 |
| MPO | 0.28 |
| IL1A | 0.28 |
| CTLA4 | 0.28 |
| APOL1 | 0.28 |
| EDN2 | 0.28 |
| KALRN | 0.28 |
| SERPINF2 | 0.28 |
| HTR2B | 0.28 |
| EDN3 | 0.28 |
| SREBF1 | 0.28 |
| ATM | 0.28 |
| TNFRSF1B | 0.28 |
| TGFB2 | 0.28 |
| VEGFB | 0.27 |
| GNRHR | 0.27 |
| STAT4 | 0.27 |
| PSMA6 | 0.27 |
| HMGB1 | 0.27 |
| F3 | 0.27 |
| ADRB2 | 0.27 |
| PCSK9 | 0.27 |
| EGR1 | 0.27 |
| BRCA1 | 0.27 |
| ADRA1A | 0.27 |
| ADRA1D | 0.27 |
| TACR3 | 0.27 |
| NOS2 | 0.27 |
| BCL2L11 | 0.27 |
| PROS1 | 0.27 |
| CYP2E1 | 0.27 |
| CDC5L | 0.27 |
| S100B | 0.27 |
| SELL | 0.27 |
| REN | 0.27 |
| PSMB9 | 0.27 |
| SMARCA4 | 0.27 |
| TERT | 0.27 |
| TLR3 | 0.27 |
| CDKN1A | 0.27 |
| NFE2L2 | 0.27 |
| GFAP | 0.27 |
| IRAK1 | 0.27 |
| EZH2 | 0.27 |
| IL7R | 0.27 |
| SELP | 0.27 |
| TEK | 0.27 |
| SDC1 | 0.27 |
| BECN1 | 0.27 |
| LPL | 0.27 |
| PDGFRA | 0.27 |
| APOC3 | 0.27 |
| PLA2G1B | 0.27 |
| GATA3 | 0.26 |
| PECAM1 | 0.26 |
| PTX3 | 0.26 |
| PARP1 | 0.26 |
| ENO2 | 0.26 |
| TTR | 0.26 |
| RIPK1 | 0.26 |
| CDK5 | 0.26 |
| LGALS1 | 0.26 |
| PRKAR1A | 0.26 |
| MMRN1 | 0.26 |
| PLAU | 0.26 |
| LPA | 0.26 |
| GRIN2B | 0.26 |
| HSPA1A | 0.26 |
| CALU | 0.26 |
| PPARGC1A | 0.26 |
| CD36 | 0.26 |
| SORT1 | 0.26 |
| PRKCE | 0.26 |
| IL9 | 0.26 |
| TXN | 0.26 |
| ANGPT1 | 0.26 |
| MAP3K5 | 0.26 |
| ATF4 | 0.26 |
| TIMP2 | 0.26 |
| MDM2 | 0.26 |
| MAP2K4 | 0.26 |
| TLR7 | 0.26 |
| SCG2 | 0.26 |
| ADAM17 | 0.26 |
| LRP1 | 0.26 |
| LDLR | 0.26 |
| FGFR1 | 0.26 |
| SELE | 0.26 |
| APOH | 0.26 |
| CYP2B6 | 0.26 |
| CYP2C9 | 0.26 |
| LCN2 | 0.26 |
| IFNB1 | 0.26 |
| CD34 | 0.26 |
| ACE | 0.26 |
| SOX2 | 0.26 |
| CALR | 0.26 |
| THBD | 0.26 |
| SOCS1 | 0.26 |
| IL33 | 0.26 |
| ALOX5 | 0.26 |
| ATG7 | 0.26 |
| ETS1 | 0.26 |
| IL4R | 0.26 |
| TRAF3 | 0.26 |
| DUSP1 | 0.26 |
| PLAT | 0.26 |
| ITGA4 | 0.26 |
| MAPT | 0.26 |
| PTHLH | 0.26 |
| PGF | 0.26 |
| GP1BA | 0.26 |
| CYP2C19 | 0.26 |
| BCL2 | 0.26 |
| CDK6 | 0.26 |
| MMP8 | 0.26 |
| CHI3L1 | 0.25 |
| IL6R | 0.25 |
| CHIT1 | 0.25 |
| AGER | 0.25 |
| HBB | 0.25 |
| PON1 | 0.25 |
| VTN | 0.25 |
| CD4 | 0.25 |
| CYBA | 0.25 |
| ADM | 0.25 |
| EFNB2 | 0.25 |
| ESR2 | 0.25 |
| CYP1A1 | 0.25 |
| ALOX15 | 0.25 |
| CTSG | 0.25 |
| PRKG1 | 0.25 |
| NAMPT | 0.25 |
| ATF3 | 0.25 |
| G6PD | 0.25 |
| PIK3CG | 0.25 |
| NRG1 | 0.25 |
| ACAA1 | 0.25 |
| RUNX1 | 0.25 |
| HSPB1 | 0.25 |
| VIP | 0.25 |
| CYP3A4 | 0.25 |
| GH1 | 0.25 |
| SQSTM1 | 0.25 |
| NLRP3 | 0.25 |
| GJA1 | 0.25 |
| LTA | 0.25 |
| INSR | 0.25 |
| PARK7 | 0.25 |
| LRG1 | 0.25 |
| MMP7 | 0.25 |
| GRIN2A | 0.25 |
| SHH | 0.25 |
| ENG | 0.25 |
| PLA2G7 | 0.25 |
| CPB2 | 0.25 |
| GRN | 0.25 |
| JUNB | 0.25 |
| PTGS1 | 0.25 |
| PRKD1 | 0.25 |
| FGF1 | 0.25 |
| XBP1 | 0.25 |
| IL10RB | 0.25 |
| GATA1 | 0.25 |
| LEPR | 0.25 |
| PLA2G6 | 0.25 |
| SLC9A1 | 0.25 |
| VIM | 0.25 |
| ROCK2 | 0.25 |
| ACSS2 | 0.25 |
| F10 | 0.25 |
| CYP2C8 | 0.25 |
| OLR1 | 0.25 |
| HLA-DRB1 | 0.25 |
| TLR5 | 0.25 |
| GPT | 0.25 |
| SNCA | 0.25 |
| ACTG1 | 0.25 |
| SREBF2 | 0.25 |
| CD69 | 0.25 |
| TRPV1 | 0.25 |
| CETP | 0.25 |
| IL1RN | 0.25 |
| SGK1 | 0.25 |
| IL10RA | 0.25 |
| TLR8 | 0.25 |
| FGFR4 | 0.25 |
| GHRH | 0.25 |
| ZEB1 | 0.25 |
| TGFBR1 | 0.25 |
| OSM | 0.25 |
| LTA4H | 0.25 |
| LIF | 0.25 |
| HSF1 | 0.25 |
| APOM | 0.25 |
| ADCYAP1 | 0.25 |
| NES | 0.25 |
| DVL1 | 0.25 |
| ANGPT2 | 0.25 |
| KLF2 | 0.25 |
| DAB1 | 0.25 |
| CACNA1D | 0.25 |
| CYP4A11 | 0.25 |
| ABCA1 | 0.25 |
| SIRT6 | 0.25 |
| HPSE | 0.25 |
| GRIA2 | 0.25 |
| NOX4 | 0.25 |
| TNFSF10 | 0.25 |
| PINK1 | 0.25 |
| HTR7 | 0.24 |
| SIRT2 | 0.24 |
| COL18A1 | 0.24 |
| NQO1 | 0.24 |
| DAPK1 | 0.24 |
| HAMP | 0.24 |
| CRHR1 | 0.24 |
| HSPB2 | 0.24 |
| NR1I2 | 0.24 |
| RNASE3 | 0.24 |
| OCLN | 0.24 |
| CECR1 | 0.24 |
| IL21 | 0.24 |
| EIF2AK3 | 0.24 |
| CD163 | 0.24 |
| TGFBR2 | 0.24 |
| NR4A1 | 0.24 |
| HTR3A | 0.24 |
| NF1 | 0.24 |
| PTGER2 | 0.24 |
| TNFSF12 | 0.24 |
| GDF15 | 0.24 |
| NTF3 | 0.24 |
| ABCB1 | 0.24 |
| NR1H3 | 0.24 |
| BSG | 0.24 |
| GLA | 0.24 |
| ARSA | 0.24 |
| VIMP | 0.24 |
| PRKCH | 0.24 |
| CYP4F2 | 0.24 |
| F9 | 0.24 |
| GSTM1 | 0.24 |
| GUCY1A2 | 0.24 |
| VASP | 0.24 |
| NEFL | 0.24 |
| CACNA1A | 0.24 |
| TNFRSF11A | 0.24 |
| TREM2 | 0.24 |
| GP6 | 0.24 |
| ADRB1 | 0.24 |
| ELN | 0.24 |
| SERPINA5 | 0.24 |
| IRAK4 | 0.24 |
| ITGA2 | 0.24 |
| COMT | 0.24 |
| CHD3 | 0.24 |
| FCGR2A | 0.24 |
| PRKAA1 | 0.24 |
| NTF4 | 0.24 |
| COL3A1 | 0.24 |
| DUSP2 | 0.24 |
| TGM2 | 0.24 |
| CD14 | 0.24 |
| LOX | 0.24 |
| TNFRSF9 | 0.24 |
| EPAS1 | 0.24 |
| SLCO1B1 | 0.24 |
| EGLN1 | 0.24 |
| IFNAR2 | 0.24 |
| FABP4 | 0.24 |
| BIRC5 | 0.24 |
| IL37 | 0.24 |
| NPPA | 0.24 |
| SIRT3 | 0.24 |
| MYLK | 0.24 |
| MMP10 | 0.24 |
| UCHL1 | 0.24 |
| ALOX12 | 0.24 |
| IL18R1 | 0.24 |
| TNFAIP3 | 0.24 |
| DPP4 | 0.24 |
| CYP2J2 | 0.24 |
| GGCX | 0.24 |
| F11 | 0.24 |
| NOTCH3 | 0.24 |
| HNF1A | 0.24 |
| PPIG | 0.24 |
| GADD45A | 0.24 |
| WRN | 0.24 |
| TNFRSF11B | 0.24 |
| PLA2G2A | 0.24 |
| SPHK2 | 0.24 |
| SOD3 | 0.24 |
| MMP12 | 0.24 |
| TGFA | 0.24 |
| PTGIR | 0.24 |
| BTG2 | 0.24 |
| IER3 | 0.24 |
| ACHE | 0.24 |
| BAK1 | 0.24 |
| BRCA2 | 0.24 |
| TP63 | 0.24 |
| ANPEP | 0.24 |
| ABCG2 | 0.24 |
| SLC6A4 | 0.24 |
| DKK1 | 0.24 |
| HLA-G | 0.24 |
| SERPINE2 | 0.24 |
| F7 | 0.24 |
| COX5A | 0.24 |
| TNFRSF12A | 0.24 |
| TNFSF14 | 0.24 |
| TAAR1 | 0.24 |
| MRAS | 0.24 |
| GRIN1 | 0.24 |
| ATF6 | 0.24 |
| GPX1 | 0.24 |
| SERPINB2 | 0.24 |
| PDGFD | 0.24 |
| UGCG | 0.24 |
| XRCC1 | 0.24 |
| USF1 | 0.24 |
| HDAC9 | 0.24 |
| SCN5A | 0.24 |
| AKT1S1 | 0.24 |
| CACNB2 | 0.24 |
| HDAC4 | 0.24 |
| ALDH2 | 0.24 |
| MBL2 | 0.24 |
| MDK | 0.24 |
| KLK1 | 0.24 |
| DLL4 | 0.24 |
| HLA-B | 0.24 |
| STK39 | 0.24 |
| CDKN2B | 0.24 |
| HLA-DQB1 | 0.24 |
| PROCR | 0.23 |
| FCGR3B | 0.23 |
| MIF | 0.23 |
| LIMK1 | 0.23 |
| CD83 | 0.23 |
| BARD1 | 0.23 |
| PDGFC | 0.23 |
| ZFP36 | 0.23 |
| GCLC | 0.23 |
| CASP7 | 0.23 |
| RBP4 | 0.23 |
| KL | 0.23 |
| NTN1 | 0.23 |
| SETD2 | 0.23 |
| CD74 | 0.23 |
| CIITA | 0.23 |
| HSPA1B | 0.23 |
| COL4A2 | 0.23 |
| OGG1 | 0.23 |
| DDIT4 | 0.23 |
| CARTPT | 0.23 |
| ZC3H12A | 0.23 |
| ABCG1 | 0.23 |
| CDKN3 | 0.23 |
| ITM2B | 0.23 |
| NR3C2 | 0.23 |
| STIP1 | 0.23 |
| CACNB3 | 0.23 |
| CDKN2C | 0.23 |
| MADD | 0.23 |
| TRPM2 | 0.23 |
| ACTA2 | 0.23 |
| COL4A1 | 0.23 |
| TRPV4 | 0.23 |
| UBQLN1 | 0.23 |
| UGT1A1 | 0.23 |
| SRF | 0.23 |
| PTGIS | 0.23 |
| UCP2 | 0.23 |
| PLIN1 | 0.23 |
| F12 | 0.23 |
| ENSG00000279576 | 0.23 |
| DEFB4A | 0.23 |
| GADD45B | 0.23 |
| PTGES | 0.23 |
| PADI4 | 0.23 |
| SERPINF1 | 0.23 |
| VDR | 0.23 |
| ADAMTS4 | 0.23 |
| PTPRG | 0.23 |
| ACP5 | 0.23 |
| SLC44A2 | 0.23 |
| CEACAM1 | 0.23 |
| TNNI3 | 0.23 |
| HAVCR1 | 0.23 |
| HMGCR | 0.23 |
| ACVRL1 | 0.23 |
| LRP6 | 0.23 |
| MYH11 | 0.23 |
| TFPI | 0.23 |
| EEF1A1 | 0.23 |
| TRPC6 | 0.23 |
| MAFB | 0.23 |
| KCNQ1 | 0.23 |
| CKB | 0.23 |
| FLG | 0.23 |
| TNFSF4 | 0.23 |
| PPP1R15A | 0.23 |
| TRIM2 | 0.23 |
| ACE2 | 0.23 |
| IL20 | 0.23 |
| CYP19A1 | 0.23 |
| HTRA2 | 0.23 |
| MSR1 | 0.23 |
| GAP43 | 0.23 |
| NR4A2 | 0.23 |
| CDH13 | 0.23 |
| PDE5A | 0.23 |
| CLDN5 | 0.23 |
| DOCK7 | 0.23 |
| CYP11B2 | 0.23 |
| RUVBL2 | 0.23 |
| IL1RL1 | 0.23 |
| ANGPTL4 | 0.23 |
| CYP3A5 | 0.23 |
| ANGPTL3 | 0.23 |
| LGALS3 | 0.23 |
| SLC6A3 | 0.23 |
| ADAMTS13 | 0.23 |
| PTPRD | 0.23 |
| NFKBIZ | 0.23 |
| CTSL | 0.23 |
| ADIPOR2 | 0.23 |
| GRIN2C | 0.23 |
| CHRNA1 | 0.23 |
| MFAP5 | 0.23 |
| PPARD | 0.23 |
| NKX2-5 | 0.23 |
| ABCC3 | 0.23 |
| TRPA1 | 0.23 |
| GPX3 | 0.23 |
| DNM1L | 0.23 |
| PAPPA | 0.23 |
| CFH | 0.23 |
| TRPM7 | 0.23 |
| NPPB | 0.23 |
| IL16 | 0.23 |
| FAF1 | 0.23 |
| EPHX2 | 0.23 |
| ABCG8 | 0.23 |
| SELPLG | 0.23 |
| TNFRSF17 | 0.23 |
| ADD1 | 0.23 |
| KCNH2 | 0.23 |
| ECE1 | 0.23 |
| ZFHX3 | 0.23 |
| PANX1 | 0.23 |
| GJA4 | 0.23 |
| ADORA2A | 0.23 |
| S100A9 | 0.23 |
| DUOX1 | 0.23 |
| DUOX2 | 0.23 |
| TBXAS1 | 0.23 |
| CA2 | 0.23 |
| NPAS4 | 0.23 |
| GSTO1 | 0.23 |
| ACOT4 | 0.23 |
| IER2 | 0.23 |
| PITX2 | 0.23 |
| MOG | 0.23 |
| RARRES2 | 0.23 |
| TNKS | 0.23 |
| GSS | 0.23 |
| P2RX7 | 0.23 |
| SERPINI1 | 0.23 |
| ACD | 0.23 |
| KRT18 | 0.23 |
| SLC1A2 | 0.23 |
| RNF146 | 0.23 |
| ASIC3 | 0.23 |
| SH2B3 | 0.22 |
| ARHGEF10 | 0.22 |
| FMO3 | 0.22 |
| TET2 | 0.22 |
| LAMP2 | 0.22 |
| PROZ | 0.22 |
| SLC18A2 | 0.22 |
| CHRNA4 | 0.22 |
| PRDX5 | 0.22 |
| HFE | 0.22 |
| ACACA | 0.22 |
| ALOX5AP | 0.22 |
| KCNJ3 | 0.22 |
| ADAMTS7 | 0.22 |
| GIG25 | 0.22 |
| CACNA1S | 0.22 |
| CACNA1F | 0.22 |
| HABP2 | 0.22 |
| CENPK | 0.22 |
| CACNA1B | 0.22 |
| RTN4R | 0.22 |
| RTN4 | 0.22 |
| P2RX4 | 0.22 |
| SLC7A11 | 0.22 |
| SAA2 | 0.22 |
| SLC5A2 | 0.22 |
| MTR | 0.22 |
| MTAP | 0.22 |
| SLC6A2 | 0.22 |
| TSPAN33 | 0.22 |
| VKORC1 | 0.22 |
| DENR | 0.22 |
| RTEL1 | 0.22 |
| LOXL1 | 0.22 |
| HSPA12B | 0.22 |
| EPHA4 | 0.22 |
| SCN2A | 0.22 |
| TNFAIP8L2 | 0.22 |
| APOD | 0.22 |
| CARD8 | 0.22 |
| KCND3 | 0.22 |
| ABO | 0.22 |
| RNF213 | 0.22 |
| LLGL2 | 0.22 |
| RENBP | 0.22 |
| CYP2D6 | 0.22 |
| HOMER1 | 0.22 |
| HOMER2 | 0.22 |
| NPC1 | 0.22 |
| ERCC4 | 0.22 |
| CCNL1 | 0.22 |
| ADA | 0.22 |
| FADS1 | 0.22 |
| G0S2 | 0.22 |
| FBLN1 | 0.22 |
| MANF | 0.22 |
| PPAP2B | 0.22 |
| NDUFC2 | 0.22 |
| AGXT2 | 0.22 |
| KCNJ1 | 0.22 |
| LGMN | 0.22 |
| HBA1 | 0.22 |
| PLA2G15 | 0.22 |
| AQP1 | 0.22 |
| MTRR | 0.22 |
| CACNG1 | 0.22 |
| FCN1 | 0.22 |
| PSRC1 | 0.22 |
| CELSR2 | 0.22 |
| AQP4 | 0.22 |
| BRAP | 0.22 |
| CA1 | 0.22 |
| PDE4D | 0.22 |
| GRIK2 | 0.22 |
| TFPI2 | 0.21 |
| CACNB1 | 0.21 |
| CACNB4 | 0.21 |
| MYBPC3 | 0.21 |
| TRPM8 | 0.21 |
| PDE11A | 0.21 |
| CACNA1I | 0.21 |
| PLAA | 0.21 |
| PMF1 | 0.21 |
| PMF1-BGLAP | 0.21 |
| RGS1 | 0.21 |
| FADS2 | 0.21 |
| CYP24A1 | 0.21 |
| CBSL | 0.21 |
| ACYP2 | 0.21 |
| LIN28B | 0.21 |
| LTC4S | 0.21 |
| PKD1 | 0.21 |
| HYOU1 | 0.21 |
| ANK1 | 0.21 |
| ALDH1A2 | 0.21 |
| GPI | 0.21 |
| NNMT | 0.21 |
| SEMA3E | 0.21 |
| MVK | 0.21 |
| SON | 0.21 |
| PC | 0.21 |
| KCNE2 | 0.21 |
| TRIB1 | 0.21 |
| AOC3 | 0.21 |
| NPR3 | 0.21 |
| HADHA | 0.21 |
| CELSR1 | 0.21 |
| ENSG00000160200 | 0.21 |
| TCN2 | 0.21 |
| NETO1 | 0.21 |
| SLC12A1 | 0.21 |
| SLC19A1 | 0.21 |
| ILF3 | 0.21 |
| PRPF8 | 0.21 |
| SMN1 | 0.21 |
| SLC8A1 | 0.21 |
| MAT2A | 0.21 |
| CCS | 0.21 |
| ABCC1 | 0.21 |
| MASP2 | 0.20 |
| FOLH1 | 0.20 |
| ABCC6 | 0.20 |
| NTNG1 | 0.20 |
| SLC12A2 | 0.20 |
| CYP2R1 | 0.20 |
| DEFB1 | 0.20 |
| DLD | 0.20 |
| ATXN2 | 0.20 |
| GCH1 | 0.20 |
| COX7A2L | 0.20 |
| COX8A | 0.20 |
| SLC12A3 | 0.20 |
| INADL | 0.20 |
| EIF5A | 0.20 |
| SPTBN5 | 0.20 |
| ALDH1L1 | 0.20 |
| ACOT7 | 0.20 |
| TRPM6 | 0.20 |
| LRRC8A | 0.20 |
| IGJ | 0.20 |
| MZB1 | 0.20 |
| PDE10A | 0.20 |
| PDE3A | 0.20 |
| MMAB | 0.19 |
| MMD | 0.19 |
| KCNJ11 | 0.19 |
| GAMT | 0.19 |
| WNK3 | 0.19 |
| WNK1 | 0.19 |
| PDE4DIP | 0.19 |
| CKM | 0.19 |
| TRPV3 | 0.19 |
| KCNIP4 | 0.18 |
| NCS1 | 0.18 |
| SRR | 0.18 |
| SLC6A8 | 0.18 |
| TSPYL6 | 0.18 |
| ZNF208 | 0.18 |
| UMOD | 0.17 |
| ABCC8 | 0.17 |
| CKMT1A | 0.16 |
| CKMT2 | 0.16 |
| ABCD2 | 0.06 |
| KIF26B | 0.06 |
| ABCD1 | 0.06 |
| KIF16B | 0.06 |

Supplementary materials 2

Supplementary material 2 is the data of 147 active ingredients in the traditional

Chinese medicine compound obtained after screening by ADMET.

| **Herb** | **TCMSP ID** | **Formula** | **PubChem CID** | **Canonical SMILES** |
| --- | --- | --- | --- | --- |
| DH | MOL004 | C22H18O10 | 6419835 | C1C(C(OC2=CC(=CC(=C21)O)O)C3=CC(=C(C=C3)O)O)OC(=O)C4=CC(=C(C(=C4)O)O)O |
| DH | MOL005 | C10H10O | 637759 | CC(=O)C=CC1=CC=CC=C1 |
| DH | MOL014 | C19H28O4 | 442377 | CC1CCCC2(C1(C(C3=C(C2)OC=C3C)OC(=O)C(C)C)C)O |
| DH | MOL016 | C15H18O7 | 11972309 | C1=CC=C(C=C1)C=CC(=O)OC2C(C(C(OC2O)CO)O)O |
| DH | MOL018 | C12H12O5 | 5319543 | CC1CC(=O)C2=C(O1)C=C(C=C2CC(=O)O)O |
| DH | MOL026 | C12H10O4 | 5315891 | CC1=CC(=O)C2=C(O1)C=C(C=C2C(=O)C)O |
| DH | MOL034 | C13H12O4 | 5319500 | CC1=CC(=O)C2=C(O1)C=C(C=C2CC(=O)C)O |
| DH | MOL035 | C15H12O4 | 24867638 | CC1=CC2C(C(=C1)O)C(=O)C3=C(C2=O)C=CC=C3O |
| DH | MOL040 | C15H12O4 | 122635 | CC1=CC(=C2C(=C1)CC3=CC(=CC(=C3C2=O)O)O)O |
| DH | MOL043 | C15H14O6 | 255538 | C1C(C(OC2=CC(=CC(=C21)O)O)C3=CC(=C(C=C3)O)O)O |
| DH | MOL048 | C19H22O8 | 5319972 | CC1=C(C(=C2C(=C1)C=CC=C2OC3C(C(C(C(O3)CO)O)O)O)O)C(=O)C |
| DH | MOL063 | C22H28O9 | 53399170 | CC1=CC(=CC(=C1)C=CC2=CC(=C(C=C2)O)OC)O.C(C1C(C(C(C(O1)O)O)O)O)O |
| DH | MOL064 | C15H14O4 | 53394021 | COC1=C(C=C(C=C1)C=CC2=CC(=CC(=C2)O)O)O |
| DH | MOL072 | C14H12O6 | 5320968 | C1C(C(=O)OC=C1O)C(=O)C=CC2=CC(=CC(=C2)O)O |
| DH | MOL082 | C20H24O9 | 11972479 | CC1=C(C(=C2C(=C1)C=C(C=C2OC3C(C(C(C(O3)CO)O)O)O)OC)O)C(=O)C |
| ZS | MOL086 | C20H20O7 | 145659 | COC1=C(C=C(C=C1)C2=CC(=O)C3=C(C(=C(C=C3O2)OC)OC)OC)OC |
| ZS | MOL089 | C9H6O4 | 5281343 | C1=COC2=CC(=CC(=C2C1=O)O)O |
| ZS | MOL090 | C20H20O8 | 358832 | COC1=C(C=C(C=C1)C2=CC(=O)C3=C(O2)C(=C(C(=C3O)OC)OC)OC)OC |
| ZS | MOL092 | C10H16 | 440917 | CC1=CCC(CC1)C(=C)C |
| ZS | MOL093 | C10H18O | 67179 | CC(=CCCC(C)(C=C)O)C |
| ZS | MOL095 | C16H13O6- | 49859576 | COC1=C(C=C(C=C1)C2CC(=O)C3=C(O2)C=C(C=C3[O-])O)O |
| ZS | MOL097 | C15H16O4 | 473252 | CC(C)C(=O)CC1=C(C=CC2=C1OC(=O)C=C2)OC |
| ZS | MOL098 | C20H20O7 | 632135 | COC1=C(C=C(C=C1)C2=CC(=O)C3=C(O2)C(=C(C=C3OC)OC)OC)OC |
| ZS | MOL099 | C19H23NO4 | 11667062 | CN1CCC23C=C(C(=O)CC2C1CC4=C3C(=C(C=C4)OC)O)OC |
| ZS | MOL102 | C16H17NO2 | 3083797 | COC1=CC=C(C=C1)CCNC(=O)C2=CC=CC=C2 |
| ZS | MOL104 | C9H14NO+ | 25202330 | C[NH2+]CCC1=CC=C(C=C1)O |
| ZS | MOL105 | C15H12O5 | 667495 | C1C(OC2=CC(=CC(=C2C1=O)O)O)C3=CC=C(C=C3)O |
| ZS | MOL113 | C15H20N2O | 7067420 | C=CCCN1CC2CC(C1)C3=CC=CC(=O)N3C2 |
| ZS | MOL114 | C10H16O2 | 53399145 | C.CCC(C1=CC=C(C=C1)O)O |
| ZS | MOL115 | C20H20O7 | 68077 | COC1=CC=C(C=C1)C2=CC(=O)C3=C(O2)C(=C(C(=C3OC)OC)OC)OC |
| HP | MOL116 | C20H26O7 | 13893598 | COC1=C(C=CC(=C1)CCCO)OC(CO)C(C2=CC(=C(C=C2)O)OC)O |
| HP | MOL117 | C6H6O2 | 14505 | CC(=O)C1=CC=CO1 |
| HP | MOL118 | C11H10O | 74128 | C1=CC=C2C=C(C=CC2=C1)CO |
| HP | MOL120 | C15H24O | 13240188 | CC1(CC2C1CCC3(C(O3)CCC2=C)C)C |
| HP | MOL121 | C15H26O | 227829 | CC1CCC(CC2=C1CCC2C)C(C)(C)O |
| HP | MOL123 | C18H18O2 | 72303 | C=CCC1=CC(=C(C=C1)O)C2=CC(=C(C=C2)O)CC=C |
| HP | MOL124 | C11H15NO2 | 46697 | CC1C2=CC(=C(C=C2CCN1)OC)O |
| HP | MOL128 | C15H26O | 6432005 | CC1=C2CC(CCC2(CCC1)C)C(C)(C)O |
| HP | MOL130 | C18H18O2 | 72300 | C=CCC1=CC(=C(C=C1)O)C2=C(C=CC(=C2)CC=C)O |
| HP | MOL131 | C12H17NO2 | 40093 | CC1C2=CC(=C(C=C2CCN1C)OC)O |
| QH | MOL136 | C20H26O4 | 25721351 | CC1(C2CCC1(C(C2)OC(=O)C=CC3=CC(=C(C=C3)O)OC)C)C |
| QH | MOL138 | C16H14O5 | 160544 | CC1(C(O1)COC2=C3C=CC(=O)OC3=CC4=C2C=CO4)C |
| QH | MOL139 | C20H26O4 | 98642978 | CC1(C2CCC1(C(C2)OC(=O)C=CC3=CC(=C(C=C3)O)OC)C)C |
| QH | MOL140 | C20H26O4 | 11724190 | CC1(C2CCC1(C(C2)OC(=O)C=CC3=CC(=C(C=C3)O)OC)C)C |
| QH | MOL141 | C17H24O3 | 6475018 | CCCCCCC=CC(C(C#CC#CC(C=C)O)O)O |
| QH | MOL143 | C9H16O | 5354833 | CCCCCCC=CC=O |
| QH | MOL145 | C10H16 | 7461 | CC1=CCC(=CC1)C(C)C |
| QH | MOL146 | C11H10 | 7002 | CC1=CC=CC2=CC=CC=C12 |
| QH | MOL147 | C10H16O | 6553876 | CC1C2CC2(CC1=O)C(C)C |
| QH | MOL148 | C10H14O2 | 101594685 | CC1(C2CC1C(=O)C=C2CO)C |
| QH | MOL150 | C16H32O2 | 21206 | CCC(C)CCCCCCCCCCC(=O)OC |
| QH | MOL151 | C15H30O2 | 151014 | CC(C)CCCCCCCCCCCC(=O)O |
| QH | MOL152 | C16H32O2 | 23618376 | CCC(C)CCCCCCCCCCC(=O)OC |
| QH | MOL154 | C17H34O2 | 22207 | CCC(C)CCCCCCCCCCCCC(=O)O |
| QH | MOL156 | C10H16 | 6654 | CC1=CCC2CC1C2(C)C |
| QH | MOL158 | C11H14O3 | 5319469 | COCC=CC1=CC(=C(C=C1)O)OC |
| QH | MOL159 | C9H18O | 13187 | CCCCCCCC(=O)C |
| QH | MOL160 | C9H14O | 19602 | CCCCCC1=CC=CO1 |
| QH | MOL162 | C12H14O5 | 735755 | COC1=CC(=CC(=C1OC)OC)C=CC(=O)O |
| QH | MOL163 | C10H18O | 11230 | CC1=CCC(CC1)(C(C)C)O |
| QH | MOL164 | C7H13 | 11115931 | CC[CH]C(=C)CC |
| QH | MOL167 | C16H32O2 | 101094508 | CC(C)CCCC(C)CCCC(C)CCC(=O)O |
| QH | MOL168 | C6H12O2 | 31256 | CC(=O)CC(C)(C)O |
| QH | MOL173 | C15H22O2 | 30942 | CCCCC#CCC#CCCCCCC(=O)O |
| QH | MOL176 | C17H19NO4 | 388787 | CN1CCC2=CCC3C(C21)C4=CC(=C(C=C4C(=O)O3)O)OC |
| QH | MOL178 | C16H14O4 | 5317436 | CC(=CCC1=C2C(=C(C3=C1OC(=O)C=C3)O)C=CO2)C |
| QH | MOL179 | C10H16 | 22311 | CC1=CCC(CC1)C(=C)C |
| QH | MOL180 | C10H16 | 7460 | CC1=CCC(C=C1)C(C)C |
| QH | MOL181 | C10H16 | 82227 | CC1=CCC2CC1C2(C)C |
| QH | MOL182 | C10H16 | 7462 | CC1=CC=C(CC1)C(C)C |
| QH | MOL187 | C12H14O4 | 10659 | COC1=C2C(=C(C(=C1)CC=C)OC)OCO2 |
| QH | MOL190 | C10H14 | 10812 | CC1=CC(=CC=C1)C(C)C |
| QH | MOL191 | C14H12O2 | 2345 | C1=CC=C(C=C1)COC(=O)C2=CC=CC=C2 |
| QH | MOL192 | C12H8O4 | 2355 | COC1=C2C=CC(=O)OC2=CC3=C1C=CO3 |
| QH | MOL193 | C11H6O4 | 5280371 | C1=CC(=O)OC2=CC3=C(C=CO3)C(=C21)O |
| QH | MOL194 | C17H16O9 | 125494 | C1=CC(=O)OC2=CC3=C(C=CO3)C(=C21)OC4C(C(C(C(O4)CO)O)O)O |
| QH | MOL195 | C15H24 | 11106485 | CC1CCC2C13CCC(=C)C(C3)C2(C)C |
| QH | MOL197 | C10H16 | 14896 | CC1(C2CCC(=C)C1C2)C |
| QH | MOL198 | C10H16 | 5281553 | CC(=CCC=C(C)C=C)C |
| QH | MOL199 | C13H20O | 539536 | CC(=O)CC1CC2CC(C1=C)C2(C)C |
| QH | MOL200 | C10H18O | 44630107 | CC1(C2CCC1(C(C2)O)C)C |
| QH | MOL201 | C12H20O2 | 44630108 | CC(=O)OC1CC2CCC1(C2(C)C)C |
| QH | MOL202 | C10H16 | 440966 | CC1(C2CCC(C2)C1=C)C |
| QH | MOL203 | C10H16O | 23308299 | CC1(C2CCC1(C(=O)C2)C)C |
| QH | MOL205 | C17H24O7 | 53399195 | CC=CC1=CC(=C(C=C1)O)O.CC1CC(CC(C1O)O)(C(=O)O)O |
| QH | MOL206 | C15H26O2 | 16085188 | CC(C)C1CCC2(C3CCC(C3C1O2)(C)O)C |
| QH | MOL207 | C10H18O | 8748 | CC(=C)C1CCC(CC1)(C)O |
| QH | MOL208 | C17H16O5 | 53398727 | CC(=CCOC1=CC(=O)OC2=C1C=C3C=COC3=C2OC)C |
| QH | MOL209 | C14H14O4 | 442104 | CC(C)(C1CC2=C(O1)C=CC3=C2OC(=O)C=C3)O |
| QH | MOL210 | C16H16O5 | 161409 | CC(=O)OC(C)(C)C1CC2=C(O1)C=CC3=C2OC(=O)C=C3 |
| QH | MOL211 | C19H20O5 | 6436246 | CC=C(C)C(=O)OC(C)(C)C1CC2=C(O1)C=CC3=C2OC(=O)C=C3 |
| QH | MOL214 | C10H16 | 440917 | CC1=CCC(CC1)C(=C)C |
| QH | MOL216 | C16H14O4 | 5316520 | CC(C)(C=C)C1=C2C(=C(C3=C1OC(=O)C=C3)O)C=CO2 |
| QH | MOL218 | C16H12O6 | 5281612 | COC1=C(C=C(C=C1)C2=CC(=O)C3=C(C=C(C=C3O2)O)O)O |
| QH | MOL227 | C10H18O | 71300291 | CC1(C2CCC(C2)(C1O)C)C |
| QH | MOL228 | C10H10O4 | 445858 | COC1=C(C=CC(=C1)C=CC(=O)O)O |
| QH | MOL230 | C5H8O4 | 53399155 | CC=CC(=O)O.C(=O)O |
| QH | MOL233 | C15H20O2 | 101821084 | CC1C2CCC(=C)C3CC=C(C3C2OC1=O)C |
| QH | MOL234 | C15H26O | 9899341 | CC1CCC(CC2=C1CCC2C)C(C)(C)O |
| QH | MOL235 | CH5N3 | 451298 | C(=N)(N)N |
| QH | MOL237 | C15H26O2 | 91704727 | CC1CCC2(C1CC3CCC2(OC3(C)C)C)O |
| QH | MOL238 | C15H26O | 21768605 | CC1=CCCC(=CCC(CC1)C(C)(C)O)C |
| QH | MOL239 | C7H16 | 524600 | CCCCCCC |
| QH | MOL240 | C6H10O | 53628050 | [CH2-]CCCCC#[O+] |
| QH | MOL242 | C16H16O4 | 14704104 | COC1=CC=C(C=C1)C(=O)OCCC2=CC=C(C=C2)O |
| QH | MOL243 | C12H8O4 | 68082 | COC1=C2C=CC(=O)OC2=C3C=COC3=C1 |
| QH | MOL244 | C10H18O | 6321405 | CC1(C2CCC1(C(C2)O)C)C |
| QH | MOL245 | C15H26O2 | 91747826 | CC(C)C1CCC(C2C1(CC(=C)CC2)O)(C)O |
| QH | MOL246 | C11H10O5 | 5318565 | COC1=C(C(=C2C(=C1)C=CC(=O)O2)OC)O |
| QH | MOL247 | C16H14O4 | 68081 | CC(=CCOC1=C2C=CC(=O)OC2=CC3=C1C=CO3)C |
| QH | MOL249 | C13H10O5 | 68079 | COC1=C2C=COC2=C(C3=C1C=CC(=O)O3)OC |
| QH | MOL250 | C12H14O2 | 5273464 | CC(C)OC(=O)C=CC1=CC=CC=C1 |
| QH | MOL251 | C11H6O3 | 10658 | C1=CC2=C(C=CO2)C3=C1C=CC(=O)O3 |
| QH | MOL255 | C10H16 | 439250 | CC1=CCC(CC1)C(=C)C |
| QH | MOL257 | C14H14O4 | 334704 | CC(C)(C1CC2=C(O1)C=C3C(=C2)C=CC(=O)O3)O |
| QH | MOL265 | C10H16 | 31253 | CC(=CCCC(=C)C=C)C |
| QH | MOL267 | C11H12O3 | 4276 | COC1=CC(=CC2=C1OCO2)CC=C |
| QH | MOL270 | C14H14O4 | 604512 | CC(C)(C1CC2=C(O1)C=C3C(=C2)C=CC(=O)O3)O |
| QH | MOL272 | C14H14O4 | 26305 | CC(C)(C1CC2=C(O1)C=C3C(=C2)C=CC(=O)O3)O |
| QH | MOL279 | C8H16O | 451254 | CCCCCCCC=O |
| QH | MOL280 | C18H34O2 | 13011408 | CCCCCCCCC=CCCCCCCCC(=O)O |
| QH | MOL282 | C14H14O3 | 5320318 | CC(=CCC1=C(C=CC2=C1OC(=O)C=C2)O)C |
| QH | MOL285 | C16H16O6 | 483513 | CC(C)(C(COC1=C2C=CC(=O)OC2=CC3=C1C=CO3)O)O |
| QH | MOL286 | C10H14 | 7463 | CC1=CC=C(C=C1)C(C)C |
| QH | MOL287 | C10H18O | 17100 | CC1=CCC(CC1)C(C)(C)O |
| QH | MOL288 | C16H14O5 | 44144315 | CC(=C)C(COC1=C2C=CC(=O)OC2=CC3=C1C=CO3)O |
| QH | MOL290 | C8H10O | 6054 | C1=CC=C(C=C1)CCO |
| QH | MOL291 | C18H18O4 | 5284444 | COC1=C(C=CC(=C1)C=CC(=O)OCCC2=CC=CC=C2)O |
| QH | MOL292 | C13H10O5 | 4825 | COC1=C(C2=C(C=CO2)C3=C1C=CC(=O)O3)OC |
| QH | MOL294 | C16H16O3 | 5320791 | COC1=CC(=CC(=C1)C=CC2=CC=C(C=C2)O)OC |
| QH | MOL297 | C10H16 | 6429260 | CC(C)C12CCC(=C)C1C2 |
| QH | MOL298 | C10H8O4 | 5280460 | COC1=C(C=C2C(=C1)C=CC(=O)O2)O |
| QH | MOL300 | C12H8O4 | 108104 | COC1=C2C(=C3C(=C1)C=CC(=O)O3)C=CO2 |
| QH | MOL305 | C10H18O | 11468 | CC(C)C1=CCC(CC1)(C)O |
| QH | MOL308 | C15H26O2 | 16046185 | CC(C)C1CCC(=C)C2CCC(C2C1O)(C)O |
| QH | MOL309 | C15H28O3 | 91884970 | CC(C)C1CCC(C2CCC(C2C1O)(C)O)(C)O |
| QH | MOL310 | C10H16 | 6451618 | CC1=CCC2(C1C2)C(C)C |
| QH | MOL311 | C10H18O | 5283345 | CCCCCCCC=CC=O |
| QH | MOL312 | C18H32O2 | 5280644 | CCCCCCC=CC=CCCCCCCCC(=O)O |
| QH | MOL315 | C9H8O3 | 637542 | C1=CC(=CC=C1C=CC(=O)O)O |
| QH | MOL316 | C13H10O8 | 129974 | COC1=C(C(=O)C2=C(C1=O)C(=C3C(=C2O)OCO3)O)OC |
| QH | MOL318 | C8H7O4- | 54675858 | COC1=C(C=CC(=C1)C(=O)O)[O-] |
| QH | MOL320 | C11H8O4 | 29920867 | C1CC(=O)OC2=C1C=C3C=COC3=C2O |
| QH | MOL321 | C15H14O4 | 66548 | CC1(C=CC2=C(O1)C=C3C(=C2OC)C=CC(=O)O3)C |
| QH | MOL322 | C14H22O2 | 5322113 | COC(=O)CCC=CCCC=CCCC=C |
| QH | MOL323 | C10H16 | 440967 | CC1(C2CCC(=C)C1C2)C |
| QH | MOL324 | C10H16 | 5320250 | CC(=CCC=C(C)C=C)C |
